# Targeting Selectivity: Improving Golgi α-Mannosidase II (GMII) Inhibitors Through In Silico Studies

**DOI:** 10.3390/biom16050680

**Published:** 2026-05-03

**Authors:** Nieves G. Ledesma, Carlos T. Nieto, Alejandro Manchado, María Ángeles Castro, David Diez

**Affiliations:** 1 Departamento de Química Orgánica, Facultad de Ciencias Químicas, Universidad de Salamanca, 37008 Salamanca, Spain; nievesgarcial11@usal.es (N.G.L.); eneas@usal.es (C.T.N.); alex92mc@usal.es (A.M.); 2 Laboratorio de Química Farmacéutica, Departamento de Ciencias Farmacéuticas, CIETUS, IBSAL, Facultad de Farmacia, Campus Miguel de Unamuno, Universidad de Salamanca, 37007 Salamanca, Spain; macg@usal.es

**Keywords:** Golgi α-mannosidase II, glycosidase inhibitors, selectivity, structure-based drug design, molecular docking, molecular dynamics, quantum mechanics, virtual screening, swainsonine

## Abstract

Aberrant glycosylation is a recognized hallmark of cancer, establishing Golgi α-mannosidase II (GMII) as strategic therapeutic target. While the natural alkaloid swainsonine demonstrated potent anticancer activity, its clinical use is hampered by toxicity from off-target inhibition of the lysosomal α-mannosidase (LMan). This review surveys computational methodologies advancing inhibitor development from empirical observations to precision structural optimization. We examine the evolution from Molecular Docking to advanced Quantum Mechanics (QM) and Molecular Dynamics (MD), highlighting their combined role in modeling metalloenzyme flexibility and energetics. Analysis reveals that selectivity relies on exploiting peripheral structural divergences, organelle-specific pH gradients, and distinct substrate conformational itineraries. In this context, electronic structure calculations and pKa predictions prove critical for designing “electrostatic switches”, inhibitors binding neutrally at Golgi pH while incurring lysosomal repulsion. Structurally, targeting the non-conserved “anchor site”, mimicking specific transition-state ring distortions and utilizing conformationally restricted scaffolds represent the most effective strategies. Integrating dynamic sampling with rigorous energetic profiling is therefore crucial for developing the next generation of safe, selective GMII inhibitors.

## 1. Introduction

Glycosylation is a fundamental cellular process through which the sequential action of glycosyltransferases (GTs) and glycosidases produces a vast diversity of glycoconjugates. These structures are an integral part of a multitude of key cellular functions, including protein quality control, cell adhesion, immune response and the regulation of metabolic and signaling pathways [1,2]. Given its critical role, aberrant glycosylation, driven by the dysregulation of these enzymes, is a pathological feature implicated in a wide spectrum of neurological, cardiovascular and autoimmune disorders [3,4,5,6,7], among others. In oncology, this phenomenon is particularly prominent and it is now established as a hallmark of cancer, often arising from altered activity in key glycosidases, such as Golgi α-mannosidase II (GMII) [8]. This enzyme, a key retaining glycosidase in the *N*-glycan maturation pathway, has emerged as a particularly attractive therapeutic target. Inhibition of GMII disrupts the formation of complex *N*-glycans, which are crucial for tumor growth, metastasis and immune evasion [9,10,11].

The natural product swainsonine, a potent GMII inhibitor (IC_50_ = 4 nM, Ki = 5 nM, hGMII, [12]) has demonstrated the aforementioned anti-cancer effects in preclinical and clinical studies, providing a strong proof-of-concept for this therapeutic strategy [13,14,15,16,17]. However, the clinical advancement of swainsonine has been severely hampered by their significant lack of selectivity. This compound also inhibits a structurally related enzyme, the human lysosomal α-mannosidase (LMan), (IC_50_ = 20 nM, Ki = 23 nM, hLMan, [12]), an enzyme with a critical housekeeping role. Off-target inhibition of LMan leads to the accumulation of unprocessed oligomannosides, inducing a phenocopy of the genetic lysosomal storage disease α-mannosidosis and causing dose-limiting toxicity [18].

Driven by the need for more selective agents, the pursuit of lead compounds with high activity and low toxicity has largely relied on structural modification. Over the past decades, the design and synthesis of various *α*-mannosidase inhibitors have explored diverse chemical architectures, primarily focusing on derivatives of swainsonine, polyhydroxypyrrolidine or polyhydroxypiperidine derivatives and disaccharide mimetics [19,20]. Despite the broad chemical space explored within these families, the challenge of selectivity remains a formidable hurdle. To overcome this, the focus of the field has shifted from traditional drug discovery toward rational, structure-based design strategies that specifically exploit the subtle structural differences between these enzymes. In this context, in silico methodologies or computer-aided drug design (CADD) have become indispensable tools. These methods allow for the detailed analysis of enzyme–inhibitor complexes at an atomic level, the prediction of binding affinities, and the proactive design of novel molecular scaffolds engineered to achieve selectivity [21,22,23].

This review will survey the pivotal computational methodologies that have been employed to address the selectivity of GMII inhibitors. The application and insights derived from a range of in silico techniques will be discussed, including molecular docking, molecular dynamics (MD) simulations, pKa predictions and advanced quantum mechanics (QM) methods such as Density Functional Theory (DFT) [24] and the Fragment Molecular Orbital (FMO) method [25]. By analyzing key case studies, we will illustrate how these computational tools are paving the way toward a new generation of potent and highly selective GMII inhibitors.

## 2. The Structural Basis for the Lack of Selectivity

### 2.1. Architecture and Catalytic Mechanism of GMII Active Site

**Figure 1:**
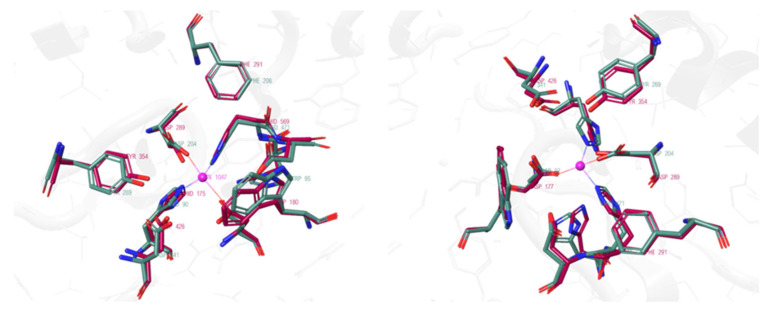
Different perspectives of the superposition between the dGMII crystal structure (PDB ID: 3BLB [29], green) and the predicted hGMII model (AlphaFold ID: AF-Q16706-F1 [27,30], magenta), shown within a 6 Å radius of the catalytic Zn^2+^ ion (pink).

Human GMII (hGMII) is a retaining glycosidase from the family of Carbohydrate-Active enZyme (CAZyme) GH38. It plays an essential role in the *N*-glycan maturation pathway by catalyzing the hydrolysis of the terminal α-1,3 and α-1,6 linked mannose residues from its GlcNAcMan_5_GlcNAc_2_ substrate, a critical step for the biosynthesis of complex-type *N*-glycoproteins [26]. To understand the structural basis of its function and inhibition, a detailed three-dimensional model of its active site is required. As no experimental structure of the hGMII has been resolved to date, structure-based design efforts have relied on high-fidelity models derived from close orthologs [27]. While several model enzymes exist, the most prominent and extensively studied surrogate is the *Drosophila melanogaster* homolog, dGMII. The widespread use of dGMII is justified by its high sequence homology with hGMII (approximately 39% [12,28]) and, critically, by the availability of numerous high-resolution X-ray crystal structures, including the pivotal complex with the archetypal inhibitor, swainsonine (PDB ID: 3BLB, [29]). Superposition of the dGMII crystal structure and the predictive model of hGMII reveals that their catalytic subsites are virtually identical, sharing the same key residues for catalysis and inhibitor binding (Table 1, Figure 1). Therefore, the following description of the GMII active site architecture is based on the foundational insights derived from the available dGMII crystal structures.

**Figure 2:**
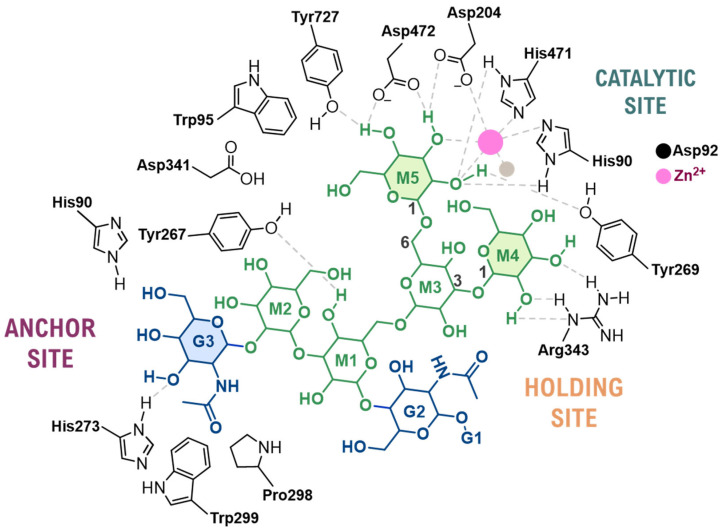
Schematic distribution of the natural substrate in the active site of dGMII. Key substrate–protein interactions are highlighted for each of the three subsites (catalytic, holding and anchor sites) that constitute the catalytic cavity.

The active site is organized into three functionally distinct subsites: the catalytic site, where hydrolysis of the glycosidic bond occurs, a holding site and an anchor site. Structural studies have revealed that most critical enzyme–substrate interactions are focused on three specific saccharide units of the natural substrate: mannose-5 (M5), mannose-4 (M4), and *N*-acetylglucosamine-3 (GlcNAc-3, G3) (Figure 2) [31].

#### 2.1.1. The Catalytic Site and Mechanism

The catalytic site is the core functional region of the active site responsible for binding the M5 mannose residue of the natural substrate to facilitate its initial hydrolysis. Within this pocket, the M5 saccharide is anchored through several key interactions: the C-2 and C-3 hydroxyls coordinate the divalent Zn^2+^ cation; a dense network of hydrogen bonds is formed with adjacent residues, including Tyr269, Tyr727, His471, and Asp472; and stacking interactions occur between the saccharide ring and aromatic residues, primarily Trp95 (Figure 2).

**Figure 3:**
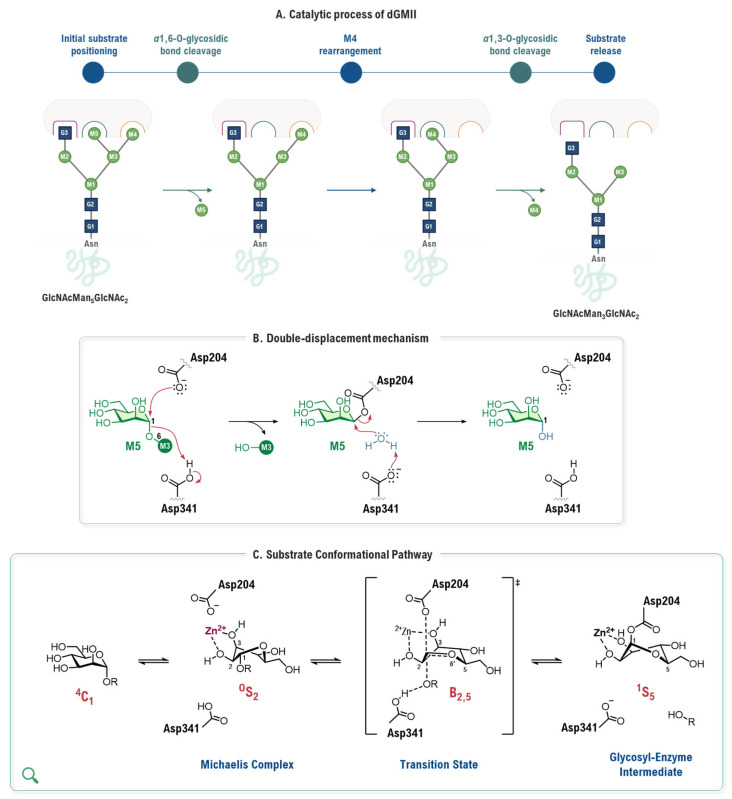
(**A**) Schematic representation of the catalytic process of dGMII (pink: anchor site; Green: catalytic site; orange: holding site. (**B**) dGMII-mediated cleavage of the α-1,6-O-glycosidic linkage. (**C**) Substrate conformational pathway, from initial coordination within the catalytic site to covalent linkage with the enzyme via the nucleophilic Asp204. Adapted from N. Shah and co-workers [31].

This precise positioning facilitates the sequential hydrolysis of two mannose residues (M5 and M4) from GlcNacMan_5_GlcNAc_2_ substrate, a process involving two cleavage events and an intermediate substrate rearrangement (Figure 3A). Each cleavage follows a two-step, double-displacement mechanism with overall retention of configuration [32]. First, the nucleophile Asp204 attacks the anomeric carbon of M5, while the general acid catalyst Asp341 protonates the leaving group, forming a covalent glycosyl-enzyme intermediate. In the second step, Asp341 acts as a general base, activating a water molecule for a nucleophilic attack that cleaves this intermediate, releasing the product and regenerating the active site (Figure 3B). This chemical process is coupled with a significant conformational distortion of the sugar ring, which is a key driver of catalysis. The substrate, initially in a stable ^4^C_1_ chair conformation, is distorted upon binding to the zinc ion and Asp204 into a strained ^0^S_2_ skew-boat (Michaelis complex), which then proceeds through a B_2,5_ boat-like transition state before forming the covalent intermediate in a ^1^S_5_ skew-boat conformation (Figure 3C) [33]. Finally, following the hydrolysis of M5, the M4 residue is repositioned into the catalytic site to undergo a mechanistically identical second cleavage event [31].

#### 2.1.2. The Holding and Anchor Sites

The holding site accommodates the M4 mannose residue, which is linked to M3 through an α-1,3-*O*-glycosidic bond. This region is located approximately 9 Å away from the nucleophilic Asp204 and is characterized by a key interaction with Arg343, which forms multiple hydrogen bonds with the M4 saccharide. Unlike the deeply buried catalytic site, the holding site is significantly more solvent-exposed. After the initial cleavage of M5, the M3 residue acts as a flexible pivot, allowing the M4 mannose to swing into the catalytic site for the second hydrolysis step. The conformational variability of M3 and the lack of restrictive residues in its binding pocket facilitate this crucial repositioning.

The anchor site is in a distinct cavity 13–14 Å away and binds the G3 residue. This subsite is critical for catalysis, serving to stabilize and orient the flexible oligosaccharide for the initial hydrolysis and tethering it to the enzyme for the subsequent processing of M4. The G3 residue is anchored through contacts with Pro298, Trp299 and His273. Furthermore, an internal “communication” network exists between the three subsites, mediated by a proton transport chain involving His273, which is located in an intermediate zone between the holding and anchor sites [31].

### 2.2. GMII and LMan: A Structural Comparison of Active Sites

**Figure 4:**
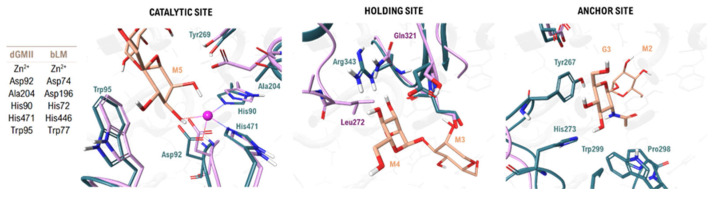
Superposition of the dGMII active site (green, PDB ID: 3CZN, [31]) with bLMan (purple, PDB ID: 1O7D, [35]), shown with the GlcNAcMan_5_GlcNAc_2_ substrate (orange) bound across the three subsites. The inset highlights key residue differences within the catalytic site.

The pivotal structural comparison between bovine lysosomal α-mannosidase (bLMan) and dGMII revealed the key differences that could be exploited for selective inhibitor design (Figure 4). While their core catalytic sites are highly conserved, evidenced by the invariant positioning of the Zn^2+^-coordinating residues (His90, Asp92, His471) and the nucleophilic aspartate (Asp204), significant divergence appears at more peripheral regions, with the overall active site of bLMan being considerably wider (20 Å, approx.) than that of dGMII (12 Å, approx.). This disparity is most pronounced in the holding and anchor subsites regions. In the holding site of dGMII, a key arginine residue (Arg343) forms strong hydrogen bonds with the substrate, whereas in the equivalent region of bLMan, this is replaced by non-interacting residues like leucine (Leu272) and glutamine (Gln321). An even greater difference is found in the anchor site, which in bLMan is a much more open and solvent-exposed cavity, lacking the specific residues required to anchor the substrate, as seen in dGMII. The functional relevance of these distal pockets was unequivocally demonstrated in a seminal study by Zhong and co-workers [34]. They showed through kinetic experiments that the truncated substrate Man_5_GlcNAc_2_, which lacks the terminal GlcNAc branch (PDB ID: 3BVX, [34]), is processed approximately 80-fold less efficiently by dGMII than the full natural substrate. The resolution of the first crystal structure of dGMII in complex with its full substrate (using a D204A mutant; PDB ID: 3CZN) [31], enabled the structural identification of the anchor site. This region is defined by two loops, absent in the structure of bLMan, which position Tyr267, Pro298, and Trp299 to create a specific hydrophobic and aromatic patch. Further analysis revealed that the GlcNAc moiety of the substrate is tightly bound through a stacking interaction with Tyr267 and a hydrogen bond with His273 [34]. Crucially, the authors noted that the lysosomal enzyme lacks an equivalent anchor site; instead, the enzyme contains a pocket of moderately conserved residues, none of which possesses the acidic or aromatic character present in the anchor site of dGMII. This structural divergence explains the preference of the lysosomal enzyme for substrates lacking the GlcNAc residue. Consequently, these two less conserved regions, the holding and anchor sites, represent the most promising “selectivity pockets” for the rational design of inhibitors that can specifically target GMII [31,35].

Beyond these structural differences, a fundamental divergence lies in the distinct proper pH of the two enzymes: GMII functions optimally at a pH of ~5.9 (in the Golgi complex), while LMan operates in the more acidic environment of the lysosome at a pH of ~4.5 [35]. This pH differential is a critical determinant of selectivity. The active sites of both enzymes contain multiple ionizable residues (Asp, Glu, Arg, His), and the inhibitors themselves often possess ionizable groups, such as the amine in iminosugars [36]. The specific protonation state of both the inhibitor and the key residues is, therefore, highly dependent on the local pH. This situation creates an opportunity to design inhibitors whose binding pattern is highly sensitive to pH, favoring a strong interaction in the milder environment of GMII, while being disfavored in the acidic medium of LMan. Computational pKa calculations and pH-dependent docking studies have become essential tools to explore and exploit these “electrostatic selectivity” strategies, as it will be discussed in later sections.

## 3. In Silico Methodologies for Targeting GMII Selectivity

Achieving inhibitor selectivity requires a rational design strategy capable of exploiting these subtle structural and electrostatic differences between the GMII and LMan active sites. With this purpose, a diverse array of in silico or CADD methodologies have become indispensable for guiding the development of the next generation of selective inhibitors. A structured summary outlining the scope and specific utility of these in silico techniques is provided in the final section, Overview of Computational Methods.

### 3.1. The Conformational Itinerary: Computational Unraveling of the Catalytic Mechanism

**Figure 5:**
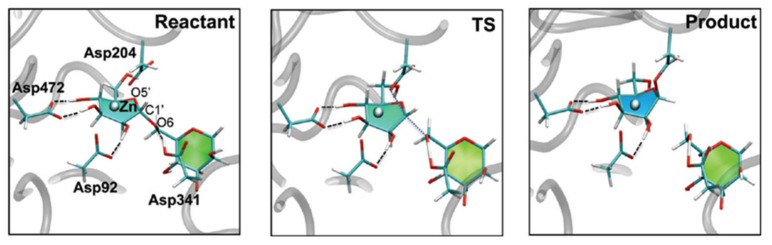
QM/MM-elucidated glycosylation pathway of dGMII. The simulation snapshots illustrate the key conformational changes in the mannose ring as it progresses from the pre-activated Michaelis complex (^0^S_2_ skew-boat) (Reactant) through a B_2,5_ boat-like transition state (TS) with significant OCI, to the final covalent ^1^S_5_ glycosyl-enzyme intermediate (Product). Zn^2+^ ligands His90 and His471 have been omitted for clarity. Reproduced with permission from Petersen and co-workers [38].

The cornerstone of rational inhibitor design is a profound understanding of the catalytic mechanism [37]. For GMII, computational studies, particularly those employing high-level QM/MM methods, have been instrumental in dissecting the intricate details of its reaction coordinate [32]. The seminal QM/MM metadynamics investigation by Petersen and co-workers is especially relevant. It provided the first detailed ab initio view of the glycosylation step, generating a free-energy landscape (FEL) of the entire reaction pathway [38]. In this work, not only is the double-displacement mechanism confirmed, but also the transition state (TS) is characterized for possessing significant oxocarbenium ion character (OCI). Critically, this work established the complete conformational itinerary of the substrate. It demonstrated that the mannose ring is forced into a pre-activated, distorted ^0^S_2_/B_2,5_ conformation upon forming the Michaelis complex with the native enzyme, before proceeding through a B_2,5_ boat-like TS (Figure 5). The simulation also provided the first mechanistic explanation for the catalytic role of the Zn^2+^ ion, proposing that it stabilizes the developing negative charge on the O2′ oxygen of the substrate during the reaction.

**Figure 6:**
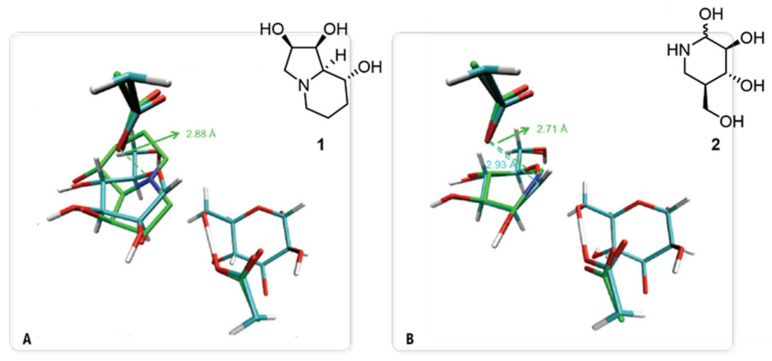
Superposition of the calculated dGMII TS with experimentally determined inhibitor complexes. The TS is compared with the bound conformations of (**A**) swainsonine (**1**) (PDB ID: 1HWW, [8]) and (**B**) **2** (PDB ID: 2ALW, [39]). Key distances for the TS (blue dashed lines) and the inhibitors (green dashed lines) are shown in Angstroms (Å) to illustrate the high degree of geometric mimicry. Reproduced with permission from Petersen and co-workers [38].

Beyond elucidating the native mechanism, this high-resolution computational model of the TS served as a powerful quantitative blueprint to evaluate the binding modes of known inhibitors. By superimposing the calculated TS structure onto experimental crystal structures of GMII-inhibitor complexes (Figure 6), the authors revealed correlations between inhibitor potency and TS mimicry. Potent inhibitors such as swainsonine (**1**) and noeuromycin (**2**) (IC_50_ = 20 µM, dGMII, [39]), for example, were remarkably successful at replicating key intermolecular distances, even if their core scaffolds differed from the substrate. The distance between the cationic center of the inhibitor and the nucleophilic Asp204 in **1** (2.88 Å) closely matched the equivalent C1′···O_Asp204_ distance in the calculated TS (2.93 Å). This analysis demonstrated that mimicking the critical intermolecular distances and charge distribution of the transition state is a key principle that informs rational inhibitor design.

**Figure 7:**
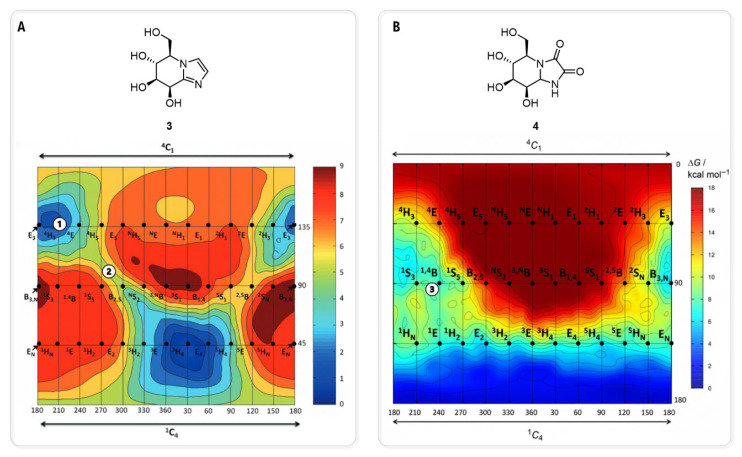
Conformational free-energy landscapes (FELs, Mercator projection), contoured at 1 kcal mol^−1^, of protonated (**3**) and kifunensine (**4**). (**A**) William and co-workers FEL of 1: **3** bound to dGMII (PDB ID: 3D4Y, [40]) in half-chair conformer; 2: **3** bound to dGMII (PDB ID 3D4Y, [40]) in boat conformer (William and co-workers) [41]. (**B**) Males and co-workers FEL of 3: **4** bound to dGMII [42] (PDB ID: 1PS3, [43]). Reproduced from R. J. William and co-workers [41,42] and A. Males and co-workers [41,42].

Building on this concept of conformational analysis, subsequent studies have employed similar ab initio metadynamics simulations to generate the conformational FEL of the inhibitors themselves. A study by R. J. William and co-workers used this approach to analyze a series of ligands, including mannoimidazole (MIm, **3**) (K_i_ = 2.0 µM, dGMII [40]) providing a quantitative basis for evaluating TS mimicry [41] (Figure 7A). Their analysis revealed that **3** is energetically poised to access multiple catalytically relevant conformations, including boat and half-chair-type conformations. Critically, by re-refining the existing crystal structure of **3** bound to dGMII (PDB ID: 3D4Y, [40]), the study provided the first direct structural evidence that the inhibitor adopts a significant population of the B_2,5_ boat conformation on enzyme. This finding was fundamental, as it validated **3** as a faithful conformational “reporter” capable of mimicking the proposed B_2,5_ transition state of the GMII catalytic pathway. Conversely, this FEL-based approach has also been used to explain inhibitor specificity, as exemplified in a study of kifunensine (**4**) by A. Males and co-workers [42] (Figure 7B). Ab initio metadynamics calculations revealed that the intrinsic low-energy conformation of **4** is a ^1^C_4_ chair. By mapping the existing crystallographic data of **4** bound to dGMII (PDB ID: 1PS3, [43]) onto this theoretical energy landscape, the authors provided a definitive rationale for its poor activity against the GH38 family (*K*_i_ = 5.2 mM, dGMII, [43]). The analysis showed that the conformation adopted by **4** within the dGMII active site, a ^1,4^B boat, while energetically accessible, represents a fundamental mismatch with the B_2,5_ boat conformation required by the GH38 catalytic itinerary. This work demonstrates how comparing experimental bound-state structures to the intrinsic conformation of inhibitor preferences can quantitatively explain family-specific inhibition.

**Figure 8:**
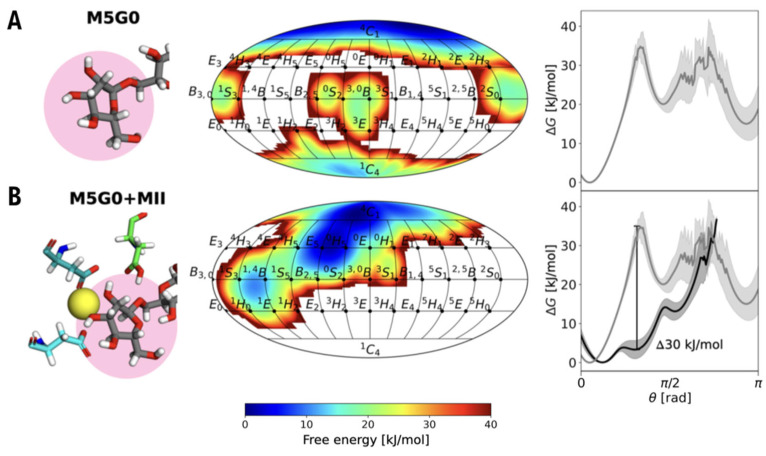
Analysis of mannose ring (M5G0) puckering in different chemical environments, as demonstrated by Grothaus and co-workers [45]. The figure shows the distortion of the terminal M5G0, monitored via the Cremer–Pople representation (2D along *ϕ* and *θ*; 1D along *θ*). Glycan carbon atoms are gray, while amino acid carbons are color-coded by residue type (light blue, Asp472; dark green, Asp92; green, Asp341. Two environments are compared: (**A**) the glycan substrate in aqueous solution (gray) and (**B**) the substrate bound within the catalytic site of GMII (M5G0 + MII) (black). Error bars, representing the standard deviation from block averaging, are shown as shaded regions. Reproduced from Grothaus and co-workers [45].

In 2025, research delved even deeper into the origins of this catalytic distortion by employing large-scale enhanced-sampling MD simulations. A groundbreaking study by Grothaus and co-workers utilized a sophisticated technique known as REST-RECT (Replica Exchange with Solute Tempering and Collective-Variable Tempering [44]) to exhaustively explore the complete conformational phase-space of the glycan substrate, both in solution and when bound to dGMII [45]. This advanced simulation method overcomes the sampling limitations of standard MD, allowing for the observation of high-energy conformational events. Their results revealed a previously unrecognized correlation between the torsional degrees of freedom of the glycosidic linkages and the puckering of the mannose ring at the catalytic site. The study demonstrates that dGMII does not simply bind pre-distorted sugar; instead, it actively induces a specific global conformation in the glycan substrate through interactions with key residues like Asp341 and the Zn^2+^ ion. This enforced torsional strain is what, in turn, drives the mannose ring (M5G0) into its catalytically required reactive state (Figure 8). This insight into the coupled, dynamic mechanism of substrate activation provides a new paradigm for inhibitor design. In this context, the development of inhibitors that combine binding to the anchor site of the enzyme with the mimicry of the conformation of the M5G0 substrate (#2*), a specific state favored by the enzyme during catalysis, represents a promising strategy. This dual-functional approach offers a powerful pathway for achieving high potency and selectivity, as it ensures tight binding to the catalytic requirements of GMII while simultaneously reducing the probability of interaction with the lysosomal isoform.

Beyond the evident utility of these computational tools, the field currently faces inherent methodological constraints. The accurate modeling of the flexible, solvent-exposed active sites of glycosidases remains a significant challenge, as the reliance on static crystal structures often neglects the conformational plasticity and the induced-fit mechanisms essential for ligand recognition. Furthermore, the simulation of the precise TS conformation and the conformational itinerary via QM/MM frameworks is often hindered by the limitations of the boundary conditions at the QM/MM interface. These factors necessitate a comprehensive interpretation of computational results within the context of the dynamic and electronic landscape of the enzyme-inhibitor interaction. For an in-depth discussion on the theoretical foundations and the broader limitations of these computational methodologies, we refer the reader to these comprehensive reviews in the field [32,33,46].

### 3.2. Harnessing Protonation and Electrostatics: A QM-Guided Path to Selectivity

A particularly insightful study in the quest for selective GMII inhibitors has emerged from a cohesive, multi-year research program led by Kóňa, Poláková and co-workers [47,48,49,50,51,52,53,54,55]. This research has systematically established and refined a powerful computational pipeline that integrates molecular docking, in situ pKa calculations, and fragment-based QM energy decomposition to move from fundamental mechanistic understanding to the prospective design of novel chemotypes. The development of this strategy is evident through a series of foundational studies that are built logically upon each other.

**Figure 9:**
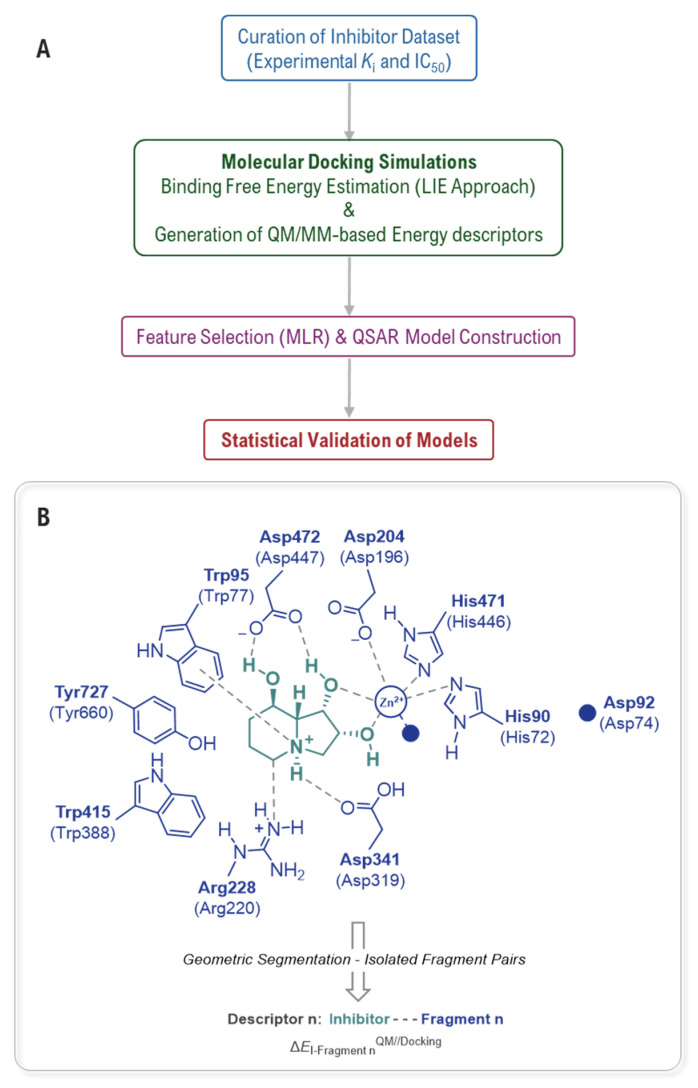
(**A**) Workflow of the computational protocol developed by Bobovská and co-workers [47]. This protocol was used for the preparation of QSAR models with QM–DFT interactions descriptors. In detail, the general scheme of the geometric decomposition used for obtaining the fragmented QM-DFT interaction energy descriptors. (**B**) Generation of QM/MM-based energy descriptors: the complex **1**-GMII was used as example, where each amino acid represented a fragment (blue: dGMII; green: **1**; gray dotted lines: diverse interactions in the complex **1**-dGMII). The numbering of amino acid residues is assigned according to crystal structures of dGMII and bLMan (the residues in round brackets). Adapted from Bobovská and co-workers [47].

The critical role of QM in achieving predictive accuracy for mannosidase affinity models was first systematically addressed by Bobovská and co-workers [47]. Faced with the failure of standard empirical Quantitative Structure-Activity Relationship (QSAR) and docking methods to reliably predict inhibitor potency for either GMII or LMan, they pioneered a pragmatic and computationally efficient hybrid empirical-QM protocol (Figure 9). The core of their strategy was to enhance classical models by incorporating fragment-based QM-DFT descriptors. This approach circumvents the prohibitive cost of a full QM treatment by calculating high-level DFT interaction energies only for key pre-selected inhibitor-residue pairs derived directly from static docked poses, assuming a rigid active site. A multiple linear regression (MLR) analysis was then employed to identify the optimal combination of empirical and QM descriptors for predicting experimental binding affinities.

For dGMII, the analysis revealed that interaction energies with Asp92, Asp204, and Arg228 were the most statistically significant QM descriptors, while for bLMan, interactions with Asp196, Arg220, Asp447, and the Zn^2+^ ion were paramount. The inclusion of these specific QM terms was transformative: the final hybrid model for GMII achieved a predictive coefficient (Q^2^) of 0.86 and successfully filtered 80% of non-active compounds, a stark improvement over the purely empirical models. This work was pivotal not only in providing a viable computational tool for lead optimization, but also in statistically, demonstrating that a small subset of specific, charge-sensitive interactions, which are poorly described by classical force fields, are the dominant drivers of binding affinity.

**Figure 10:**
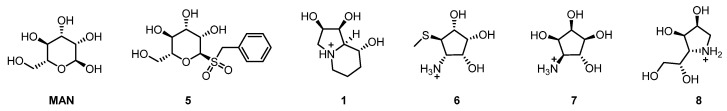
Chemical structures of the ligands studied by Sládek and co-workers in their protonated forms: mannose (MAN), mannose derivative L5 (**5**), swainsonine (**1**), mannostatin A (MSA, **6**), MSA synthetic analog L5 (**7**) and MSA synthetic analogue L6 (**8**) [48].

Paradigmatic study by Sládek and co-workers leveraged QM to resolve the fundamental question of the active protonation state of GMII inhibitors [48], providing a deep physical rationale for the predictive importance of these residues. Examining mannose (MAN), its derivative L2 (**5**) (IC_50_ = 2 mM, dGMIIb, [56]), **1**, mannostatin A (MSA, **6**) (IC_50_ = 0.15 µM, dGMIIb, [56]), and synthetic analogs L5 (**7**) and L6 (**8**) (64% inhibition, at 1 mM, hGMII, [17]) each in both their protonated and deprotonated states (Figure 10), their multi-layered analysis began with SAPT (Symmetry Adapted Perturbation Theory [57]) calculations of the diverse ligand···(D1, D2, D3, D4) complexes, which revealed a complex interplay of forces governing recognition.

**Figure 11:**
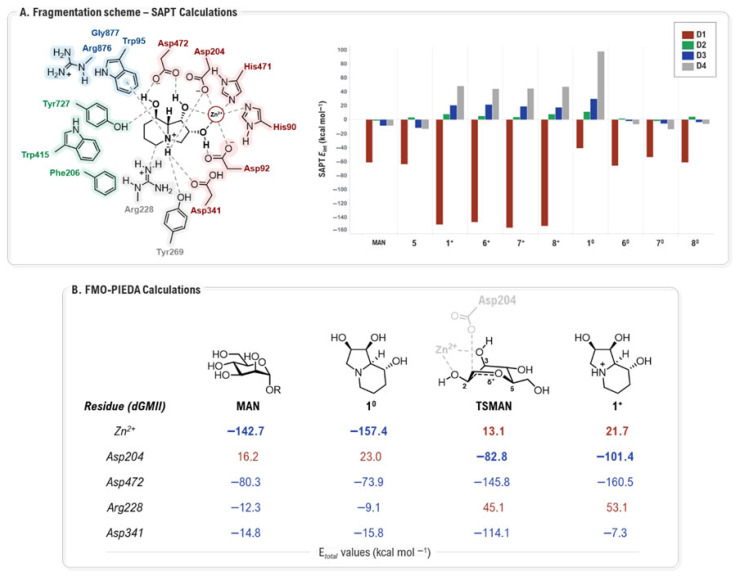
(**A**) SAPT fragmentation scheme and interaction energies. (**Left**): representation of the **1**-dGMII active site complex as example showing the four domains used in SAPT calculations (Red residues: D1; green residues: D2; blue residues: D3; gray residues: D4). (**Right**): SAPT interaction energies (E_int_) for ligand···domain complexes across all ligands in both neutral (L^0^) and protonated (L^+^) states. Symbol ^+^ means that the compound is charged positively and symbol ^0^ means that the compound is neutral (**B**) FMO-PIEDA per-residue interaction energy decomposition (E_total_) of MAN, **1^0^**, TSMAN and **1^+^** with selected residues (blue: attractive interaction energy; red: repulsive interaction energy). Adapted from Sládek and co-workers [48].

This approach established that, while the core D1 domain (Figure 11A, red residues) provides the dominant stabilizing energy for all ligands, the peripheral domains act as highly specific filters. The hydrophobic D2 domain (Figure 11A, green residues), for instance, was found to be almost universally unfavorable for inhibitors, regardless of their charge state, due to a dominant exchange-repulsion term that punishes suboptimal steric fit, a penalty not paid by the perfectly adapted natural substrate, MAN. More strikingly, the D3 (Figure 11A, blue residues) and D4 domains (Figure 11A, gray residues) were identified as protonation-sensitive electrostatic switches; they became strongly repulsive specifically towards protonated inhibitors due to unfavorable charge–charge interactions with basic residues Arg876 (D3) and Arg228 (D4) (Figure 11A). To dissect this phenomenon at an atomic level, the study then employed FMO-PIEDA (Pair Interaction Energy Decomposition) analysis [58,59] to quantify the contributions of individual residues within the crucial D1 domain. This identified the molecular origin of the observed “pattern switch” with high precision. For neutral inhibitors, the interaction pattern closely mimicked that of MAN, with the catalytic Zn^2+^ ion providing the primary attractive force. In contrast, for protonated inhibitors, the overall interaction with the Zn^2+^ ion became repulsive due to a global electrostatic clash. This energetic loss was critically compensated by a dramatic strengthening of attractions to the surrounding acidic residues, particularly Asp204, which flipped its role from repulsive (towards neutral ligands) to strongly stabilizing. Ultimately, by integrating these findings, the study concluded that the interaction signature of the protonated inhibitor mirrors that of the transition state (TSMAN) of the enzymatic reaction (Figure 11B). This provides a robust, quantum-level explanation for the high potency of these compounds and powerfully demonstrates how the delicate balance between steric fit, electrostatics, and a protonation state of the inhibitor collectively dictates the binding affinity and mechanism.

**Figure 12:**
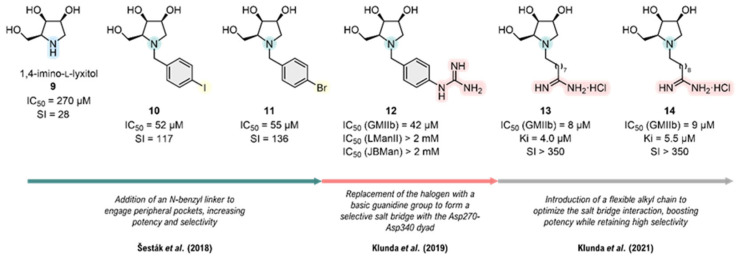
A Computation-Guided strategy for the stepwise optimization of selective GMIIb inhibitors based on the *N*-substituted 1,4-imino-L-lyxitol scaffold (**9**). The scheme illustrates the progression from a simple core (**9**) to highly potent and selective derivatives (**13** and **14**) through key structural modifications, including the addition of *N*-linkers and terminal basic groups [51,52,53].

The first application of this integrated strategy was demonstrated by Šesták and co-workers in a computation-guided design of *N*-benzyl substituted pyrrolidines [51]. The design was based on a molecular docking hypothesis that a *N*-benzyl linker could occupy a peripheral, non-conserved pocket to confer selectivity. Crucially, in situ pKa calculations indicated that these new *N*-benzyl derivatives, unlike the unsubstituted 1,4-imino-L-lyxitol (**9**) (IC_50_ = 270 µM, GMIIb) (Figure 12) or **1**, bind preferentially in their protonated form to both GMII (pH 6.0) and LMan (pH 4.5). This study also revealed the intricate plasticity of the active site, showing that the ionization state of adjacent residues like Asp340 is highly sensitive to both the specific bound inhibitor and the protonation state of the main catalytic acid, Asp341. Subsequent FMO-PIEDA analysis provided a detailed quantitative rationale. The calculations revealed that steric hindrance displaces the core of the inhibitor, significantly weakening the electrostatic interaction with the catalytic nucleophile Asp204; the E_es_ value dropped from −156 kcal·mol^−1^ for the unsubstituted core **9** to a range of ≈ −90 to −110 kcal·mol^−1^ for the *N*-benzyl substituted derivatives, **10** and **11** (Figure 12). This loss is compensated by a complex balance of forces, including strong electrostatic repulsion from Arg228 (+50 kcal·mol^−1^) and Arg876 (+30 kcal·mol^−1^). These repulsions are overcome by powerful dispersion forces, dominated by the interaction with Trp95 (E_disp_ ≈ −30 to −40 kcal·mol^−1^). Critically, the benzyl linker itself establishes stabilizing dispersion interactions with the repulsive arginine residues and with Tyr269, anchoring it in the peripheral cavity. This computational model successfully guided the synthesis of the first micromolar inhibitors, **10** (IC_50_ = 52 µM, GMIIb) and **11** (IC_50_ = 55 µM, GMIIb), with a significant selectivity index (>100-fold) (Figure 12).

Building on this strategy, Klunda and co-workers refined the design by functionalizing the *N*-benzyl linker with terminal basic groups [53]. Their approach was guided by a previous molecular docking hypothesis established by Poláková and co-workers [55] that aimed to form a highly specific salt bridge with a non-conserved polar dyad, Asp270-Asp340, unique to the dGMII active site. As predicted, compound **12** (Figure 12) with a guanidinium group optimally positioned by its linker proved to be the most potent one (IC_50_ = 42 µM, K_i_ = 19 µM, GMIIb). Furthermore, pKa calculations supported the hypothesis that these dual-basic inhibitors would likely exist in different protonation states at the distinct pH environments of the Golgi and the lysosome, providing an additional layer to the selectivity mechanism and successfully validating the targeting of a specific, non-conserved subsite (Figure 12).

A subsequent study by Klunda and co-workers further explored this concept by replacing the *N*-arylalkyl linker with a more flexible *N*-alkyl chain, also capped with a basic group [52]. While originating from a similar docking hypothesis targeting the Asp270-Asp340 dyad, the most profound insight of the study came from a synergistic combination of NMR spectroscopy and high-level DFT calculations. This combined approach was essential to analyze the conformational preferences of the inhibitor and protonation behavior. The DFT calculations revealed that the puckering of the pyrrolidine ring is directly coupled to its protonation state; for instance, the neutral form prefers an *E*_3_ or *E*_4_ envelope conformation, while the protonated form flips to a ^4^*E* or *E*_4_ conformation. Supported by experimental NMR data, these computational findings led to a sophisticated, pH-dependent model where the inhibitors with two ionizable groups are proposed to bind to GMII (at pH 6.0) in their neutral form and its associated conformation, but as a dicationic species with a rearranged ring geometry to LMan (at pH 4.5). This design strategy proved highly effectiveness. For instance, the derivatives **13** (IC_50_ = 8 µM, GMIIb) and **14** (IC_50_ = 9 µM, GMIIb) (Figure 12) emerged as the most potent inhibitors of the series, exhibiting a K_i_ of 4.0–5.5 µM and an exceptional selectivity index (SI > 350) (Figure 12). This work demonstrates that selectivity arises not just from differing interactions, but also from the complex interplay between the protonation state of the inhibitor and its intrinsic conformational preferences.

**Figure 13:**
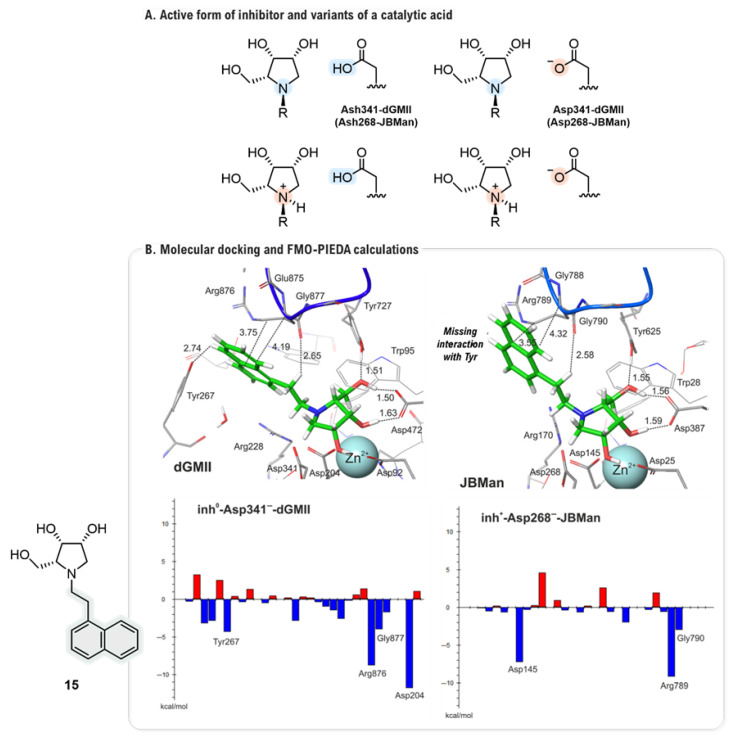
(**A**) Schematic representation of the different protonation states of the inhibitor and the catalytic aspartate predicted to be active in dGMII versus JBMan. (**B**) FMO-PIEDA interaction energy decomposition (blue: attractive: red: repulsive) for the *N*-linker of **15** (green) in both dGMII (**right**) and JBMan (**left**). Reproduced with permission from Kóňa and co-workers [49].

The investigation was extended to a new series of highly selective imino-d-lyxitols, the enantiomers of the l-lyxitol scaffolds studied in prior works [51,52,53]. A particularly deep mechanistic analysis by Kóňa and co-workers employed a full computational pipeline to explain the origins of their potent and selective activity [49]. The study began by tackling the complex interplay between the binding pose of the inhibitor, its protonation state, and the ionization of key catalytic residues (Asp341 in dGMII and Asp268 in *Jack Bean* α-mannosidase, JBMan). Using geometries from both docking and extensive QM/MM optimizations, a detailed pKa analysis was performed. The analysis uncovered a complex scenario: while all inhibitors were predicted to bind in their protonated form to JBMan (calculated pKa ≈ 7.4–8.6 at pH 4.5), the situation in dGMII was highly dependent on the precise binding conformation. Tighter binding poses, with shorter distances to the Zn^2+^ ion, favored a neutral inhibitor form (calculated pKa ≈ 4.6–5.7 at pH 6.0), a state also influenced by the ionization of the catalytic acid Asp341, which itself was found to exist in a pH-dependent equilibrium between its neutral (Ash^0^) and ionized (Asp) forms (Figure 13A). FMO-PIEDA calculations were performed on the most relevant charge states for each enzyme, using the highly potent nanomolar inhibitor **15** against *Caenorhabditis elegans* GMII (AMAN-2) (IC_50_ = 210 nM, K_i_ = 150 nM) as representative example (Figure 13B) in order to determine which structural features were responsible for this selective binding. The analysis quantified that the interaction energy of the *N*-linker (Δ*E*_linker-E_) was significantly more favorable in dGMII than in JBMan (e.g., −34.19 kcal·mol^−1^ versus −15.16 kcal·mol^−1^ for inhibitor **15**) (Figure 13B). This large energy difference was attributed to the specific engagement of the linker with a peripheral loop (Glu875-Arg876-Gly877) and, critically, the aromatic side chain of Tyr267 in dGMII (the active site of JBMan lacks a corresponding tyrosine). Therefore, the high selectivity of the d-scaffold was attributed to a powerful dual mechanism: a favorable neutral protonation state in the Golgi combined with the unique and strong anchoring of its linker to a non-conserved aromatic residue.

**Figure 14:**
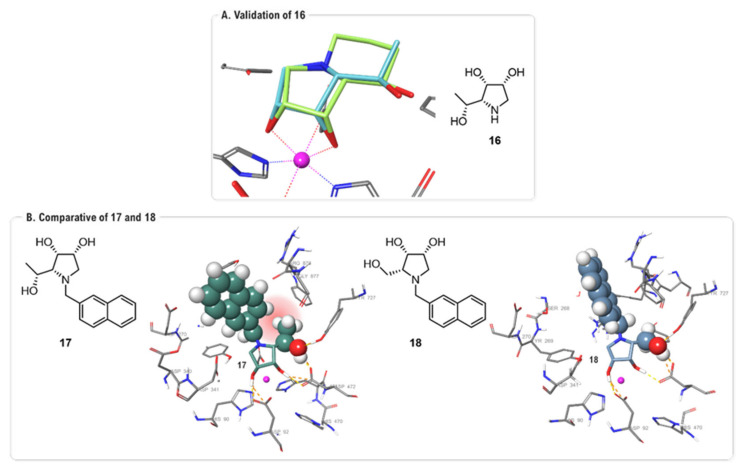
Rational design and analysis of C-5 substituted inhibitors. (**A**) Superposition of inhibitor **16** (blue, docked) and swainsonine (green, PDB ID: 3BLB [29]) within the dGMII active site. (**B**) A structural comparison between inhibitor **17** and analog **18**. The model reveals a direct intramolecular steric clash (red shadow) in **17** between the bulky moieties. Adapted from Kalník and co-workers [54].

In 2023, a study by Kalník and co-workers explored a novel design strategy by modifying the 1,4-imino-D-lyxitol core at the C-5 position [54]. A (*R*)-1-hydroxyethyl group was introduced at the C-5 position of the five membered ring to mimic the second ring of **1** (Figure 14A). FMO-PIEDA analysis validated this approach, quantifying a substantial attractive energy of -49.3 kcal·mol^−1^ for this C-5 moiety in the most potent inhibitor, 6-deoxy-1,4-dideoxy-1,4-imino-D-mannitol (6-deoxy-DIM, **16**) (IC_50_ = 0.24 μM, AMAN-2). This strong interaction was attributed to two key hydrogen bonds with Tyr727 and Asp472. Calculations also rationalized why concurrent *N*-alkylation was detrimental to activity by comparing inhibitor **17** (IC_50_ = 22 μM, AMAN-2) with a previously synthesized analog, **18** (IC_50_ = 0.24 μM, AMAN-2) [49]. An intramolecular steric clash between the bulky C-5 and *N*-substituents in compound **17** was found to disrupt the optimal binding of the core of the inhibitor, thus explaining its reduced potency (Figure 14B).

**Figure 15:**
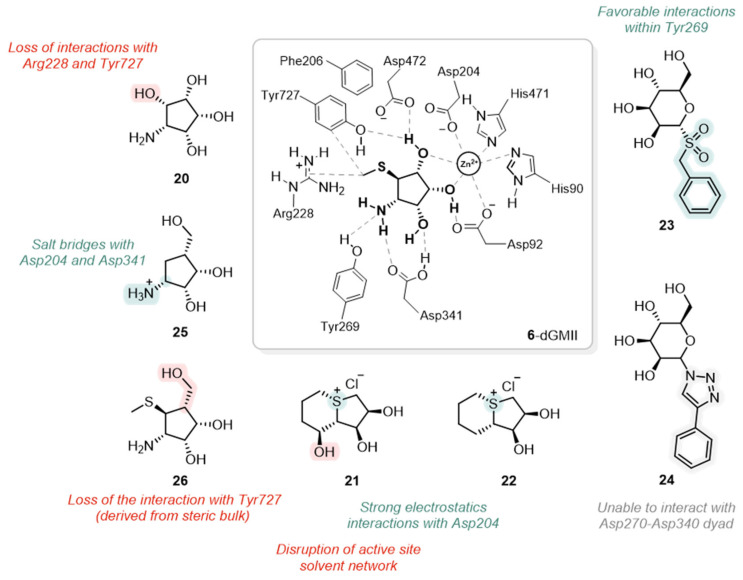
Interactions of representative docked GMII inhibitors. Central panel: binding mode of **6** showing zinc coordination and key interactions. Compounds **20**–**26** illustrate structural modifications affecting binding affinity. Color annotations indicate favorable (green) or unfavorable (red) interactions [55,56,67,68,69,70].

This research program culminated in a 2024 study by Kalník and co-workers, where the strategy was applied to rationally modify **1** [50]. The design, guided by molecular docking, introduced a (5*S*)-benzyl substituent hypothesized to induce a slight conformational distortion, this strain being more pronounced in the lysosomal enzyme (Figure 15). In situ pKa calculations pointed to a divergent recognition mechanism again, with a complex protonation equilibrium in GMII versus a single protonated state in LMan (Table 2). FMO-PIEDA analysis provided the definitive rationale for the success of the inhibitor **19**, confirming the role of the C-5 benzyl group as a steric driver for selectivity. Calculations showed that this group contributes minimally to binding energy (~5%), but it forces a core distortion that is energetically more costly in the lysosomal enzyme. This elegant mechanism explained the exceptional outcome: a nanomolar inhibitor (**19**, K_i_ = 23 nM, AMAN-2) with an outstanding selectivity index of 870.

The literature reviewed here indicates that GMII inhibitor selectivity cannot be rationalized solely by steric complementarity. High-level QM methodologies (DFT, SAPT and FMO-PIEDA) are required to describe electronic polarization and electrostatic switching effects neglected by classical force fields, demonstrating that pH-dependent in situ protonation, and thus the electronic state of the inhibitor is a key determinant of selectivity alongside molecular topology. However, it is critical to acknowledge that the current methodology for assessing the pKa of ligands in Golgi-resident metalloenzymes often relies on a hierarchical approach. First, DFT calculations are used to establish the intrinsic pKa of the ligand in aqueous solution; the close agreement between experimental (pKa ~7.4, [60]) and calculated (pKa ~7.8, [48]) values for **1**, among other iminosugars [48], confirms the robustness of the quantum mechanical baseline. Subsequently, empirical calculations, such as PROPKA [61], or more rigorous approaches based on continuum electrostatics, like the Poisson-Boltzmann-based H++ server [62,63], are employed to estimate the pKa shifts in the inhibitor within the enzymatic receptor. While this operational balance is essential for computational feasibility, it faces inherent limitations in metal-coordinated active sites, where the polarization induced by the Zn^2+^ ion is often poorly captured [64,65]. Moreover, translating these predictions to in cellulo and in vivo contexts remains challenging; the Golgi and lysosomal microenvironments are not merely defined by the bulk pH, but are influenced by the local ionic strength, the macromolecular crowding, and the competitive ion binding, which likely modulate the protonation of the inhibitor in ways currently oversimplified by in silico models [66]. Recent NMR studies have provided valuable evidence in this regard, revealing that the pyrrolidine ring of *N*-substituted inhibitors may undergo distinct protonation states at the Golgi (pH 6.0) versus the lysosomal (pH 4.5) pH [52], confirming that these microenvironments modulate the electronic state of the inhibitor. Despite this, such experiments in polar solution do not fully account for the sub-organellar complexities that current in silico models often oversimplify. Moving forward, the integration of hybrid QM/MM frameworks, capable of explicitly modeling both the dynamics of the metal center and the complex sub-organellar environment, will be essential to bridge the gap between in silico predictions and clinical efficacy, effectively shifting the computational bottleneck from empirical discovery toward precision structural optimization.

### 3.3. Targeting Structural Divergence: Using Molecular Docking to Exploit Peripheral Sites

As established in Section 2.2, the significant structural divergence between GMII and LMan in the peripheral holding and anchor sites provides the primary rational basis for achieving inhibitor selectivity. Consequently, a major focus of CADD efforts has been to design inhibitors with chemical moieties capable of productively engaging these “selectivity pockets”. Molecular docking has been the principal computational tool used to generate and evaluate hypotheses for targeting these non-conserved regions.

In 2004, an early and seminal study that applied molecular docking to this problem was conducted by Li and co-workers, who used it in conjunction with ab initio conformational analysis to rationalize the SAR of non-azasugar inhibitors like mannostatin A (**6**) [67]. Their computational models explained why **6** (K_i_ = 0.21 μM, hGMII) is a markedly more potent inhibitor than its simpler analog, aminocyclopentitetrol (**20**) (Figure 15) (K_i_ = 50 μM, hGMII). The docking studies predicted that, while both compounds occupied the catalytic site, the unique thiomethyl group of **6** provided a crucial advantage by establishing favorable hydrophobic interactions with peripheral residues Arg228 and Tyr727, an unavailable interaction for **20**. Furthermore, the docking models successfully rationalized a counter-intuitive SAR upon *N*-benzylation. For simpler core **20**, the added benzyl group as analogue **23** improved potency by forming a stabilizing stacking interaction with Tyr269 without energetic penalty (Figure 15). In contrast, for **6**, the models predicted that achieving the same stacking interaction required a conformational tilt of the core of the inhibitor, which disrupted its key hydrogen bonds with the active site, thus decreasing its overall affinity.

Molecular docking also serves as a critical tool for generating structural hypotheses that guide deeper experimental investigation. This is powerfully illustrated in a study by Kumar and co-workers, targeting two nearly identical sulfonium-ion inhibitors, **21** (IC_50_ = 2 mM, dGMII) and **22** (IC_50_ = 14 µM, dGMII) (Figure 15) with 140-fold great difference in potency [68]. While initial docking simulations identified several plausible binding modes, the static models could not account for this dramatic activity gap. This discrepancy between prediction and experimental evidence prompted a high-resolution X-ray crystallographic analysis of both complexes, which revealed a more subtle mechanism. The weaker inhibitor **21** was found to induce a minor shift in Arg228 that displaced a key water molecule, disrupting the entire active site solvent network, an effect not caused by the more potent analog **22** (Figure 15). This work exemplifies the synergy between computational prediction and experimental validation, where resolving discrepancies with initial docking models can lead to the discovery of sophisticated selectivity drivers, such as the critical role of the solvent structure.

Building on this approach, a study by Poláková and co-workers provided a more targeted example of docking use to guide the design of selective inhibitors [56]. The main goal of their study was to modify the aglycone unit of α-D-mannose derivatives to improve selectivity towards GMII. Since the α-D-mannose core itself shows no inhibitory activity, any observed effects could be directly attributed to the newly introduced anomeric substituents. Their design focused on replacing the anomeric oxygen with a sulfonyl group linked to an arylalkyl moiety, and molecular docking was employed to rationalize their findings. Computational models provided a crucial hypothesis for the selectivity of the most successful compound, benzylsulfonyl derivative **23** (Figure 15). The models predicted that its benzyl linker forms specific, favorable interactions within a peripheral pocket, most notably with the side chain of Tyr267 (not conserved in the lysosomal enzyme), pinpointing this specific ligand-residue interaction as a key structural determinant of selectivity. This work was significant as it computationally identified the Tyr267 pocket as a specific and druggable “selectivity hot-spot”, providing a clear target for subsequent rational design efforts.

Identification of another critical “selectivity pocket” came from a subsequent foundational study by the same group in 2015, which computationally laid the groundwork for a highly successful series of later inhibitors [55]. After observing that series of 1,2,3-triazole-linked mannoses were inactive against GH38 family enzymes, molecular docking was used to rationalize this finding. The docking models not only predicted a poor binding mode for the triazole linker but, more importantly, provided a key strategic insight. The analysis explicitly identified a non-conserved pocket (termed site B3 in the original study) formed by the negatively charged Asp270-Asp340 dyad in dGMII (neutral Asn262-Ser318 pair in the lysosomal homolog). The models showed that triazole-based inhibitors, e.g., derivative **24** (Figure 15), completely failed to engage with this potentially selective subsite. This study was first to computationally highlight the Asp270-Asp340 dyad as a specific, druggable target, implicitly proposing a forward-looking design strategy that, as will be discussed in Section 2.2, would later be successfully exploited to achieve high selectivity.

The power of molecular docking in rationalizing strict stereochemical requirements for inhibition is well demonstrated in a study by Zajičková and co-workers on novel carbasugar analogs [69]. After synthesizing a series of 4a-carba-D-lyxofuranose derivatives, they observed that only a single stereoisomer, 1-amino-4a-carba-*β*-D-lyxofuranose (**25**) (Figure 15), was active (IC_50_ = 200 µM, GMIIb). The computational models offered a clear atomistic explanation for this stark SAR. The docking poses of the inactive compounds revealed a geometric mismatch, where their stereochemistry prevented key interactions with the Zn^2+^ ion and surrounding aspartates. In contrast, the model of the active inhibitor **25** showed a perfect fit, mimicking the binding mode of **6**. Crucially, its protonated amino group was ideally positioned to form a bidentate salt bridge, an “ionic clamp”, with both the catalytic nucleophile Asp204 and the general acid/base Asp341, an unique interaction to this specific stereoisomer (Figure 15).

In a related study on **6** analogs, Kalník and co-workers investigated the effect of replacing key functional groups [70]. While the resulting compounds, such as homomannostatin A (**26**) (Figure 15), retained selectivity for dGMII, their potency was significantly diminished. Molecular docking successfully elucidated the structural basis for this loss of activity. The models predicted that the bulkier hydroxymethyl group of **26** introduces steric hindrance within the active site, forcing the cyclopentane ring of the inhibitor into a different puckered conformation (^3^*E* vs. ^5^*E* for **6**). This conformational rearrangement was shown to disrupt favorable contacts with peripheral residues, like Tyr727 (Figure 15). The study therefore established a valuable negative design principle, highlighting the delicate steric and conformational balance required at this position for potent inhibition.

**Figure 16:**
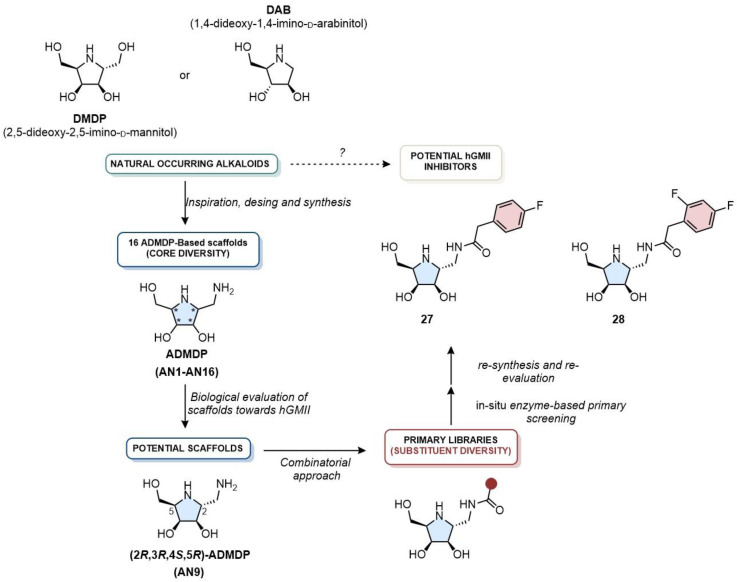
Rational design approach for hGMII inhibitors inspired by pyrrolidine-based natural products (DMDP and DAB). Adapted from Cheng and co-workers [72].

A landmark research program from Cheng and co-workers [12,71,72,73], spanning for nearly a decade, exemplifies a complete cycle of modern drug discovery methodology, from initial scaffold identification to a successful in vivo candidate. The computational journey began with a foundational 2013 natural alkaloid (e.g., 2,5-dideoxy-2,5-imino-D-mannitol, DMDP, or 1,4-dideoxy-1,4-imino-D-arabinitol, DAB) inspired study that systematically explored all sixteen stereoisomers of aminodeoxy-DMDP (ADMDP) (Figure 16) [72]. Through a combination of synthesis, screening, and homology modeling/molecular docking studies, they identified **27** (IC_50_ = 0.3 µM, hGMII) and **28** (IC_50_ = 0.5 µM, hGMII) as promising micromolar inhibitors of hGMII (Figure 16). The docking models revealed that the unique (2*R*,3*R*,4*S*,5*R*) configuration of **27** allowed it to perfectly mimic the key coordinating interactions with the Zn^2+^ ion and surrounding aspartates, while its C-2 aminomethyl group projected into a solvent-exposed region, marking it as an ideal vector for future diversification.

**Figure 17:**
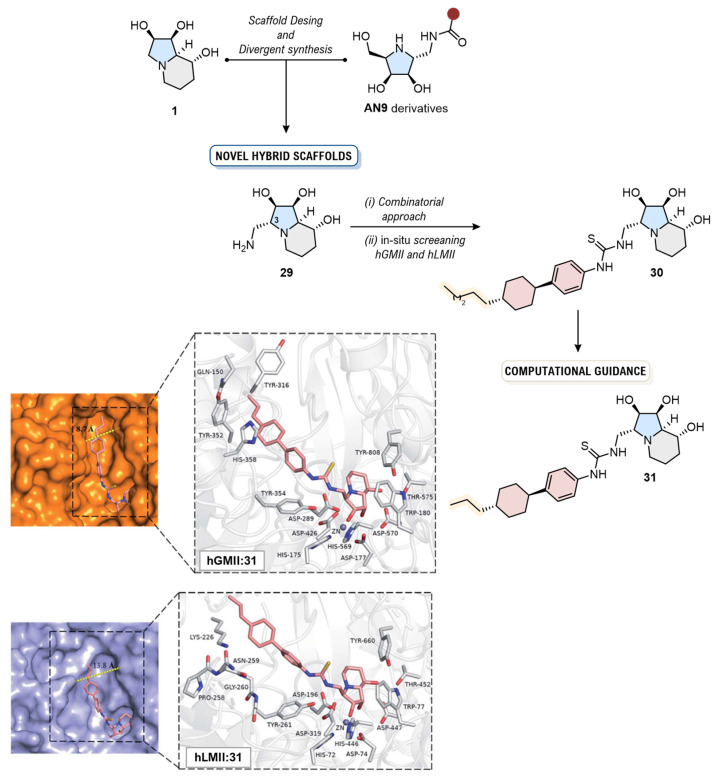
Computation-Guided Synthesis (CGS) strategy for the optimization of the selective hGMII inhibitor, **31**. Reproduced from Cheng and co-workers [12].

Building directly on this finding, a subsequent landmark study by the group aimed to address the primary limitation of **27**, its conformational flexibility [12]. Their innovative approach began with a “Natural Product-Inspired Combinatorial Chemistry” (NPICC) phase, starting with a carefully considered scaffold design. Inspired by the rigid bicyclic skeleton of **1** and the validated **27** core, the authors designed a novel, bicyclic iminosugar scaffold, **29** (Figure 17) (K_i_ = 0.097 µM, hGMII). This new core was engineered to combine the conformational rigidity of **1** with the strategically placed aminomethyl group of **27**. This scaffold then served as the core for a 96-membered library, and an in situ enzymatic screening identified a primary hit, **30** (K_i_ = 0.35 µM, hGMII), with a promising 13.5-fold selectivity.

This hit then entered a “Computation-Guided Synthesis” (CGS) phase for rational optimization. Molecular docking models provided a clear rationale for its selectivity: the docking pose revealed that the 4-hexylcyclohexyl moiety at the C-3 position of the inhibitor fits tightly into a well-defined hydrophobic pocket in hGMII, an interaction disfavored in the wider, more hydrophilic active site of hLMan. Critically, the models also identified a suboptimal π–π stacking interaction with Tyr354, guiding the in silico design of a new analog, **31**. The subsequent synthesis and evaluation of **31** afforded an outstanding result: a nanomolar competitive inhibitor of hGMII (K_i_ = 30 nM) with a remarkable 106-fold selectivity over hLMan (K_i_ = 3200 nM). Subsequent in vivo studies in a hepatocellular carcinoma mouse model confirmed that **31** exhibited significant anti-tumor activity comparable to sorafenib [74], without inducing the accumulation of oligomannosides, thus overcoming the dose-limiting toxicity of **1**. This study stands as a landmark achievement, showcasing how a synergistic pipeline from systematic scaffold exploration to precise, computation-guided lead optimization can successfully overcome the long-standing challenge of achieving GMII selectivity (Figure 17).

**Figure 18:**
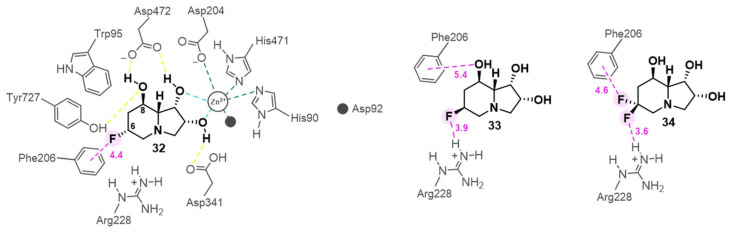
Interactions with dGMII (PDB ID: 1HWW, [8]), (6*R*)-fluoro-D-swainsonine (**32**), (6*S*)-6-fluoro-D-swainsonine (**33**), 6,6-difluoro-D-swainsonine (**34**). Only the interactions that differ from the common interaction pattern observed in **32** are shown for **33** and **34**; conserved contacts have been omitted for clarity. Adapted from Gao and co-workers [20].

In 2025, molecular docking has been employed to guide the subtle yet powerful strategy of fluorination [20], a particularly well-suited tactic for sterically constrained positions. This fact is significant, as prior SAR studies established the C-6 position of **1** as intolerant to substitution [75]. A study by Gao and co-workers addressed this limitation by demonstrating that the small size of fluorine allows it to be accommodated [20]. Their molecular docking models provided a clear rationale for why the C-6 fluorinated analogs (**32**, **33** and **34**; IC_50_ = 0.50 µM, 0.78 µM, 0.44 µM, JBMan) (Figure 18) successfully retained the high potency of **1**. Models predicted that fluorine atom acts as an effective isostere of hydrogen [76], fitting into the tight peripheral pocket with minimal steric perturbation. Crucially, the analysis revealed that the fluorine atoms were not passive placeholders but actively contributed to binding depending on their stereochemistry: the axial fluorine (as in **32** and **34**) establishes stabilizing anion–π interactions with Phe206, whereas the equatorial fluorine (as in **33** and **34**) forms hydrogen bonds with Arg228 (Figure 18).

In summary, the case studies presented in this section highlight the multifaceted role of molecular docking as a cornerstone of rational inhibitor design for GMII. It has proven to be an indispensable tool for generating initial design hypotheses, rationalizing complex and even counter-intuitive SARs, and identifying key “hot-spots” for selectivity, such as the Tyr267 pocket and the Asp270-Asp340 dyad. However, these studies also underscore that docking is more powerful when its static predictions are critically evaluated against experimental data, as discrepancies can often unveil more subtle and dynamic mechanisms of recognition [77], such as the crucial role of solvent networks or ligand strain [78].

### 3.4. Virtual Screening for Novel Hit Discovery

Beyond the optimization of known scaffolds, virtual screening (VS) offers a powerful strategy for de novo hit discovery by searching vast chemical libraries. For GMII, however, this approach has proven to be particularly challenging due to the complex nature of its active site [65,78,79]. The studies in this section narrate the evolution of VS strategies, from early setbacks that highlighted these difficulties to the development of sophisticated to multi-stage pipelines that have ultimately proven to be successful.

A foundational study by Englebienne and co-workers was among the first ones to report on a prospective virtual screening campaign against GMII and starkly illustrated involved challenges [78]. The study began by rigorously evaluating the performance of several common docking programs (including GOLD [80], FlexX [81], and Glide [82]) to determine their ability to reproduce the crystallographic poses of known inhibitors. After this initial benchmarking phase (covering both self- and cross-docking), Glide was identified as the most accurate tool, particularly in modeling the crucial coordination around the active-site Zn^2+^ ion. Further validation of Glide for a prospective screening scenario was performed by the conduction of an enrichment study, demonstrating that Glide could successfully distinguish a set of 10 known active compounds from a much larger library of 1.000 decoy molecules. Half of the active compounds were retrieved in the top 10% regardless of the selected protonation states, although the variation in the initial recovery rate, with between 3 and 5 compounds found in the top 2% depending on the states chosen. However, despite these promising validation results, the study is perhaps more recognized for its critical analysis of the inherent difficulties in modeling the GMII active site. The authors concluded that the failure of standard, rapid docking protocols to afford active compounds in de novo campaigns stemmed from several key challenges: the proper handling of the electrostatic and coordination geometry of the catalytic Zn^2+^ ion, the crucial role of conserved structural water molecules that mediate hydrogen bond networks and the inherent flexibility of the active site.

**Figure 19:**
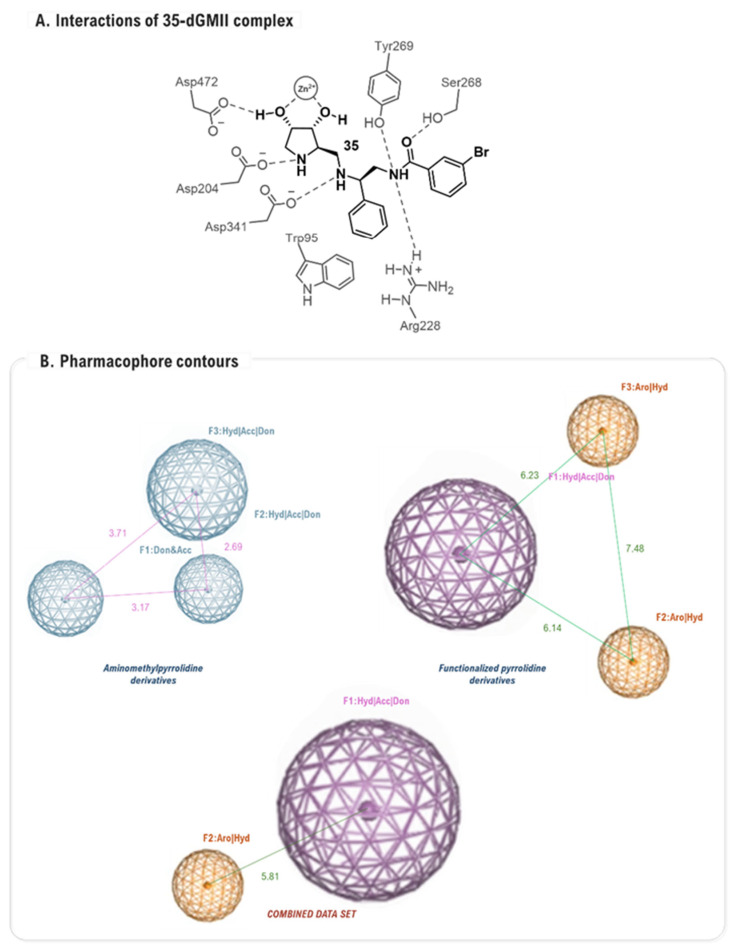
(**A**) Predicted binding mode of the most potent compound **35** within the active site of dGMII. (**B**) Pharmacophore contours derived from the focused library. (**Top**) Model for the aminomethylpyrrolidine 3,4-diol series and functionalized pyrrolidine derivatives. (**Bottom**) Consensus model for the combined dataset. Adapted from Moorthy and co-workers [83].

Moorthy and co-workers performed a combined VS, QSAR, and pharmacophore study on a focused library of 30 pyrrolidine derivatives, comprising aminomethylpyrrolidine 3,4-diol derivatives and functionalized pyrrolidines [83], demonstrating that docking could be effective in a more constrained and targeted context. In this work, the goal was not de novo discovery but rather to rationalize SAR and validate a computational model. Using AutoDock 4.2 [84], the molecular docking protocol successfully ranked the inhibitors, with predicted binding energies establishing well correlation with experimental activities. The analysis of the top-ranked inhibitor, compound **35** (89% inhibition at 1 mM, JBMan), revealed a detailed map of interactions including hydrogen bonds with Asp204 and Tyr269, and π–π stacking with Trp95 (Figure 19A). These insights were then abstracted into a 3D pharmacophore model that defined the essential spatial arrangement of chemical features required for potent inhibition. Specifically, the model highlighted the importance of two hydrophobic/acceptor/donor (Hyd/Acc/Don) features and one aromatic/hydrophobic (Aro/Hyd) feature, with key distances between them calculated to be in the range of 5.8 to 7.5 Å (Figure 19B). This study served as an important proof-of-concept, demonstrating that a synergistic approach combining docking with ligand-based models could successfully rationalize the SAR of a compound series.

**Figure 20:**
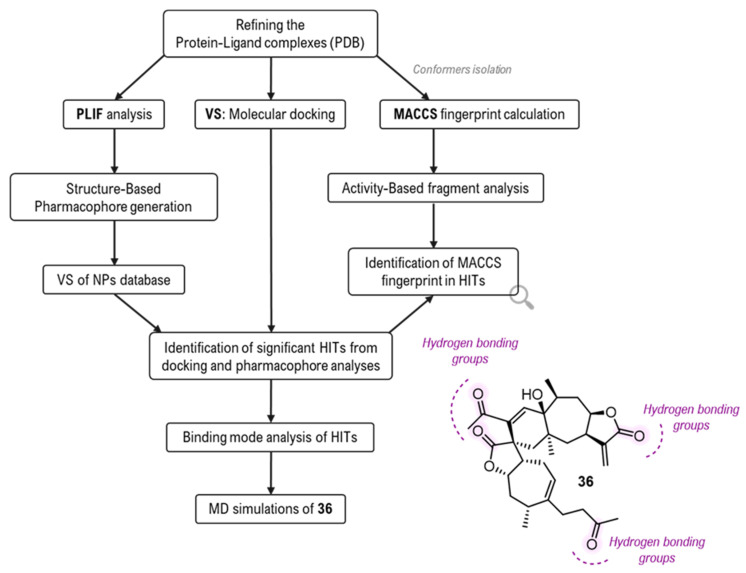
Hierarchical virtual screening workflow developed by Moorthy and co-workers. The flowchart illustrates the integrated computational strategy combining PLIF-based pharmacophore modeling, molecular docking, and MACCS fingerprint analysis to identify potential inhibitors. The chemical structure of the resulting hit, **36**, is displayed with its important structural features. Adapted from Moorthy and co-workers [85].

The first breakthrough in applying VS for genuine hit discovery targeting the orthosteric site came in 2021, employing a more sophisticated strategy. A study by Moorthy and co-workers utilized a structural bioinformatics pipeline to screen a curated collection of natural products (NPs) [85]. This workflow integrated molecular docking with a structure-based pharmacophore model derived from Protein-Ligand Interaction Fingerprints (PLIF) [86] of available crystal complexes. Additionally, the authors employed molecular access system (MACCS) fingerprint analysis to statically pinpoint specific structural fragments [87], such as aromatic rings and nitrogen atoms, that correlate with favorable inhibitory activity (Figure 20). Following docking into dGMII active site (PDB ID: 1HWW, [8]) and in silico ADMET filter to assess drug-likeness, the study identified Pungiolide C (**36**) as a potent hit (IC_50_ of 0.5 µM). Detailed analysis, validated by MD simulations, revealed that this novel scaffold stabilizes within the active site through hydrogen-bonding networks with residues such as Arg228, Tyr267 and Asp341. Notably, the MD trajectory revealed that even though **36** shifted away from Zn^2+^ ion, the complex remained highly stable due to extensive hydrophobic contacts with Trp95. Crucially, the in silico ADMET filter confirmed their drug-like potential, predicting optimal lipophilicity for good bioavailability and a favorable metabolic profile.

**Figure 21:**
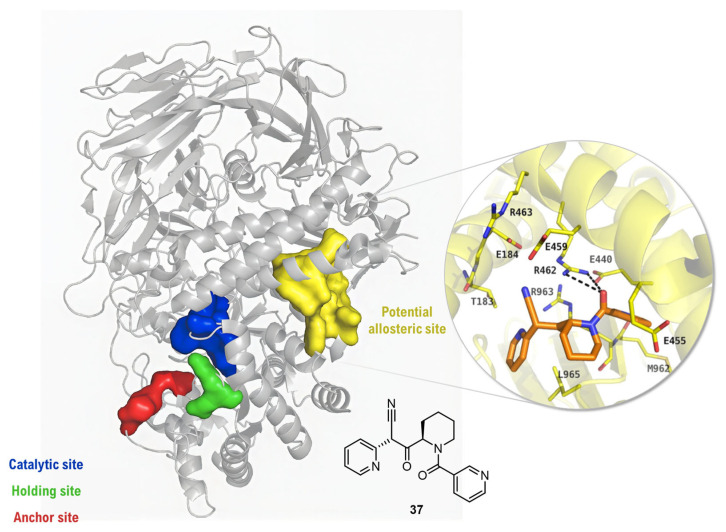
Structural overview highlighting the spatial separation between the canonical active site subsites (blue, green, red) and the novel allosteric cavity (yellow), with the inset displaying the predicted binding mode of hit **37** interacting with residues Arg462 (R462) and Glu459 (E459). Reproduced from Irsheid and co-workers [88].

Representing a landmark paradigm shift, a study by Irsheid and co-workers pioneered a VS strategy that circumvented the challenge of active-site homology by identifying and targeting a novel allosteric site [88]. Their workflow began by using the Site Finder algorithm to discover a previously uncharacterized [89], druggable pocket on the dGMII surface, approximately 30 Å away from the catalytic center (Figure 21) Evaluation with DoGSiteScorer [90] confirmed the high druggability of this pocket (score 0.83) compared to the polar active site. In order to mine a library of over 5.5 million compounds, a hierarchical pipeline was then implemented. This process initiated with a physicochemical filtering for lead-like properties, followed by a structure-based pharmacophore search, tailored to the features of the new pocket (specifically targeting Glu459 and Arg462), which reduced the candidate pool to ~41,000 molecules. These compounds were then subjected to a large-scale molecular docking campaign with DOCK 3.6 to predict binding affinities. This in silico cascade successfully identified a novel hit scaffold, compound **37**, which was subsequently confirmed experimentally as a micromolar, non-competitive inhibitor (IC_50_ ≈ 217 µM, dGMII). This study was pivotal as it provided the first proof-of-concept for a computationally driven strategy to achieve GMII inhibition by targeting a non-conserved allosteric site, offering a powerful new avenue for achieving inhibitor selectivity.

These modern hierarchical strategies, which combine ligand- and structure-based methods with subsequent computational filters like ADMET profiling, have now proven to be a viable and powerful approach for the discovery of novel, non-obvious scaffolds for GMII inhibition from diverse chemical libraries. In conclusion, the application of VS to GMII illustrates a clear learning curve. The initial challenges posed by a complex metalloenzyme active site have been progressively overcome by shifting from simple, single-step docking to sophisticated, multi-stage computational pipelines.

### 3.5. Molecular Dynamics Simulations: Capturing Flexibility and Stability

**Figure 22:**
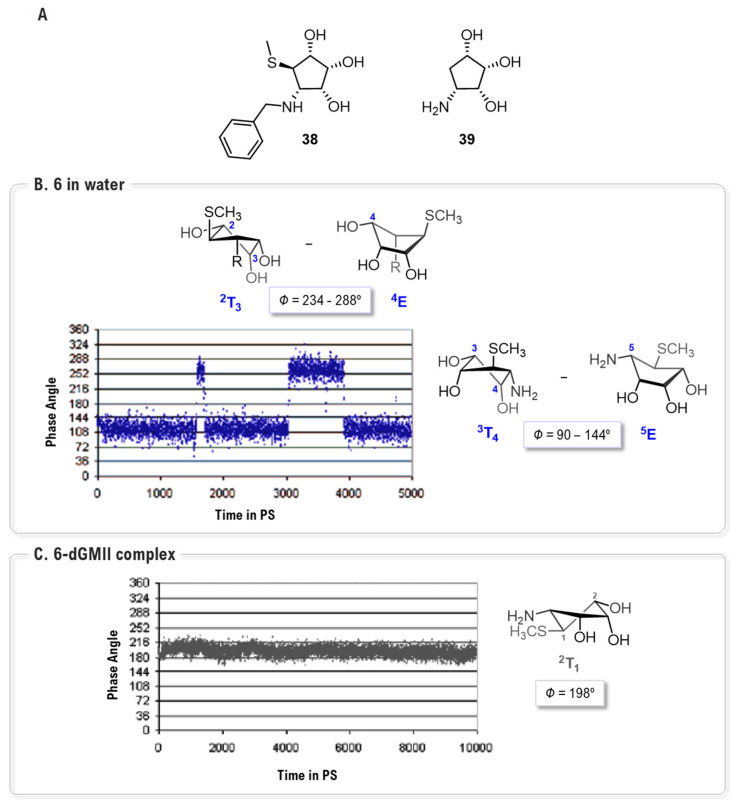
(**A**) Structure of compounds **38** and **39**. (**B**,**C**) Time-dependent evolution of phase angles (*Φ*) from MD simulation trajectories for compound **6**. (**B**) **6**-solvated in water. (**C**) **6**-bound to dGMII. In aqueous solution (**B**), **6** populates two distinct clusters: the first consists of conformers between ^3^T_4_ and ^5^E (90–144°) and the second includes conformers between ^2^T_3_ and ^4^E (234–288°). In contrast, the bound conformation (**C**) of the five-membered ring differs from the populations observed in the free state. Upon binding, the ring is restricted to a specific conformational family centering around the ^2^T_1_ conformation (≈201°) throughout the trajectory. Reproduced with permission from Kawatkar and co-workers [91].

One of the earliest studies applying Molecular Dynamics (MD) simulations to a GMII inhibitor was conducted by Kawatkar and co-workers on **6** and its analogs, **38** and **39** [91] (Figure 22A). Their work provided crucial dynamic insights into the structural basis of its high potency. MD simulations of the free ligand revealed its intrinsic flexibility, showing that its five-membered ring dynamically populates two main conformational families (pseudorotational itineraries between ^3^T_4_ and ^5^E and conformers between ^2^T_3_ and ^4^E). Critically, simulations of the enzyme-inhibitor complex demonstrated an induced-fit binding mechanism, confirming that **6** adopts a higher-energy ^2^T_1_ conformation upon binding (Figure 22B,C). For instance, the analysis showed that crucial hydrogen bonds, such as those between the 2-hydroxyl group of the inhibitor and Asp472, were maintained during the entire simulation.

This computational approach also successfully rationalized the SAR of the series. MD simulations explained the lower potency of **38** (K_i_ = 0.249 µM) by revealing a stable intramolecular S···H-Ar interaction that rendered the molecule too rigid to form productive contacts with the enzyme. Conversely, the analysis highlighted the importance of the thiomethyl group, absent in the dramatically weaker inhibitor **39** (K_i_ = 265 µM) by showing that it establishes a complex network of stabilizing interactions, including a key non-bonded contact with the Arg876 backbone and crucial hydrophobic interactions within a peripheral pocket formed by Phe206 and Tyr727. This foundational work exemplifies how MD can be used to validate binding modes, quantify interaction stability and explain complex SAR trends.

Prior to the widespread use of long-timescale MD simulations, early attempts to add a layer of dynamic and experimental validation to static docking models often employed a combination of NMR and computational techniques. A pioneering example of this approach was presented by Wen and co-workers [92], who established a combined Saturation Transfer Difference (STD) NMR/Molecular Modeling protocol [93]. Although this study did not include explicit MD simulations, its logic, using computational models to interpret and reproduce dynamic experimental data (the saturation transfer effect), laid the foundational groundwork for more sophisticated MD-based validation protocols that would follow.

**Figure 23:**
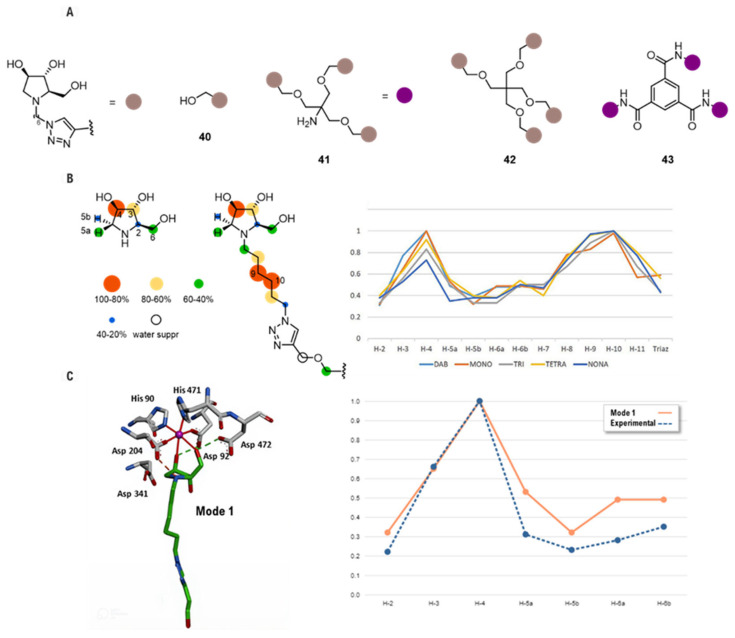
(**A**) Structures of the multivalent library. (**B**) Epitope mapping by STD NMR. Left: The heat-map indicates the protons in closest contact with the protein surface (orange, 100–80%). Right: Comparison of STD profiles for all compounds reveals an identical interaction pattern. (**C**) Computational modeling of the ligand-enzyme complex. (**Left**): Proposed binding mode showing the ligand **40** coordinating the Zn^2+^ ion within the active site. (**Right**): Validation of the model through the strong correlation between calculated (orange solid line) and experimental (blue dashed line) STD intensities. Reproduced with permission from Mirabella and co-workers [94].

Expanding on this concept of integrating NMR with computational modeling, a later study by Mirabella and co-workers replaced the static docking models with full MD simulations to create a more powerful and dynamic synergistic protocol to elucidate the binding mode of a series of multivalent pyrrolidine inhibitors, **40–43**, (Figure 23A) in the absence of crystal structure [94]. The study began with STD NMR experiments [95] to generate an “epitope map”, which experimentally identified the specific inhibitor protons in direct contact with the enzyme (Figure 23B). Several binding hypotheses were then generated via manual docking and subsequently refined through 20 ns MD simulations to identify the most stable pose. An excellent agreement was found between this in silico predicted spectrum using CORCEMA-ST [96] and the experimentally measured data, providing high confidence in the proposed atomic model of the complex [97]. Notably, while the core binding mode was conserved (Figure 23C), the inhibition assays revealed that these multivalent pyrrolidine inhibitors exhibited a remarkable selectivity for GMIIb over the lysosomal isoform (LManII) due to cross-linked enzyme aggregates. The multivalent effect was significant for GMIIb, with the nonavalent derivative **43** emerging as the most potent compound, showing a potency over 700 times greater than its monovalent counterpart against this enzyme. This affinity enhancement was notably absent for LManII, suggesting that the overall architecture of GMIIb, but not the lysosomal enzyme, can productively engage with multivalent scaffolds.

**Figure 24:**
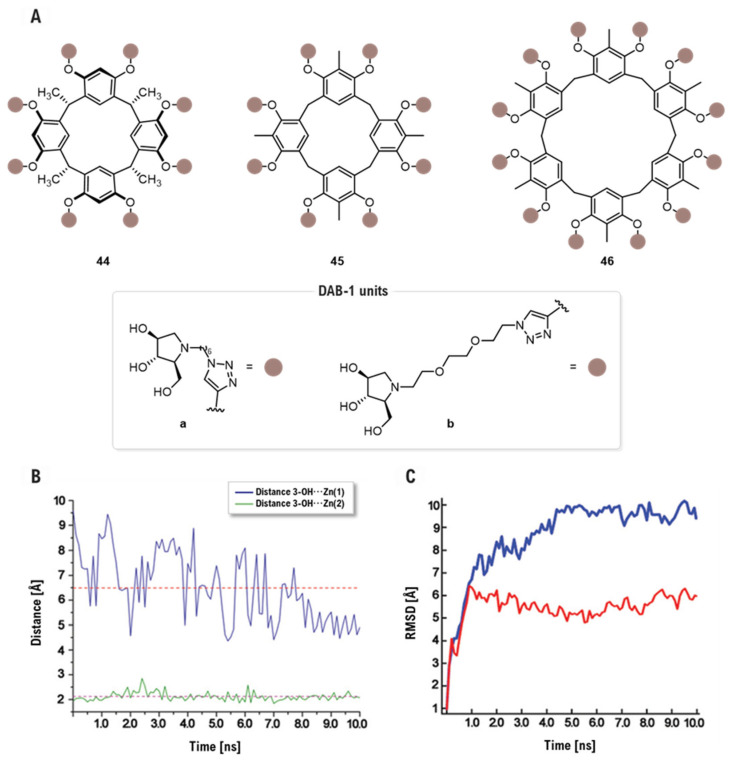
(**A**) Structures of the octavalent and dodecavalent resorcinarene clusters, **44–46**, decorated with DAB-1 units (**a**: DAB–1 moiety directly linked to the triazole; **b**: DAB–1 moiety connected to the triazole via a flexible diethylene glycol linker). (**B**) Analysis of the binding mechanism for the dodecavalent ligand **46a** within the active site of the enzyme (JBMan). The plot monitors the distance between the hydroxyl groups of the ligand and the catalytic Zn^2+^ ions during MD simulation. The stable trajectory (green line, ~2.12 Å) indicates strong chelation to one Zn^2+^ ion, while the fluctuating distance (blue line, ~6.5 Å) confirms that the ligand binds only one metal ion at a time. (**C**) RMSD analysis highlighting the impact of scaffold preorganization. The significantly lower deviation of the rigid *C*-methylated derivative **44a** (red line) compared to the flexible analog **45a** (blue line) demonstrates higher conformational stability, minimizing the entropic penalty upon binding. Reproduced with permission from Della Sala and co-workers [98].

Further advancing the integration of computational modeling with multivalent design, a subsequent study by Della Sala and co-workers explored resorcinarene macrocycles, **44–46**, as scaffolds for DAB inhitopes [98] (Figure 24A). In contrast to the cross-linking and aggregation mechanism proposed by Mirabella [94], MD simulations revealed that these clusters enhance affinity through a statistical rebinding mechanism, as their global size prevents simultaneous chelation of multiple active sites. By monitoring Zn⋯DAB-1 distances, researchers observed that, while one unit chelates a Zn^2+^ ion at ~2.12 Å, the dimensions of the scaffold keep the next closest unit at a non-coordinating distance of ~6.5 Å. The dodecavalent derivative **46a** emerged as the most potent inhibitor, exhibiting an exceptional 1114-fold selectivity for GMIIb (IC_50_ = 0.7 μM) over the LManII (IC_50_ = 780 μM) (Figure 24B). Furthermore, RMSD analysis during MD confirmed that structural preorganization is crucial; the rigid scaffold **44a** (IC_50_ = 3.7 μM, GMIIb) showed lower conformational fluctuations and higher efficiency in anchoring to the protein than the flexible **45a** (IC_50_ = 5.3 μM, GMIIb). Notably, NCI (*Non-Covalent Interactions*) and NBO (*Natural Bond Orbital*) [99,100] calculations clarified that hydrophilic linker in **46b** (IC_50_ = 28.5 μM, GMIIb) reduce potency due to electrostatic repulsions with Arg789, whereas the hydrophobic chains in 5a establish stabilizing ^γ^C-H⋯O=C interactions [101] with the same residue (Figure 24C).

**Figure 25:**
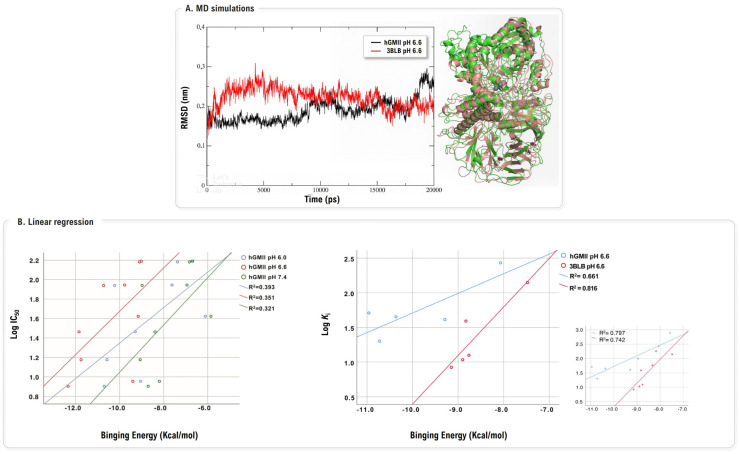
(**A**) RMSD analysis of active site residues during 20 ns MD simulations at pH 6.6 for hGMII (black line) and dGMII 3BLB (red line). Right: Superposition of the equilibrated hGMII (green) and dGMII 3BLB (salmon) structures. (**B**) Linear regression analyses correlating predicted binding energies with experimental inhibitory activities. (**Left panel**): Correlation for Klunda compounds (dGMII-optimized inhibitors) [31,33] tested in hGMII at different pH values. (**Right panel**): Correlation for Armstrong compounds (hGMII-validated inhibitors) [70] for both hGMII (blue line) and dGMII (red line) at pH 6.6. Inset: Expanded correlations after correcting suspected experimental data assignment errors. Reproduced with permission from: “Golgi α-Mannosidase: Opposing Structures of Drosophila Melanogaster and Novel Human Model Using Molecular Dynamics Simulations and Docking at Different pHs”, by Drogalin and co-workers, published in *Journal of Biomolecular Structure and Dynamics*, 42(5), 2714–2725, 2024. Copyright © Taylor & Francis. Reprinted by permission of Informa UK Limited, trading as Taylor & Francis Group [102].

The application of MD has more recently evolved to proactively refine and validate the protein models themselves, a critical step for improving predictive accuracy. A 2024 study by Drogalin and co-workers directly challenged the long-standing practice of using dGMII as a direct surrogate for the hGMII [102]. The authors first constructed a new homology model of hGMII [103], which was then subjected to extensive 100 ns MD simulations followed by 20 ns simulations of enzyme-inhibitor complexes at various pH values (6.0, 6.6 and 7.4) to achieve a structurally realistic and equilibrated state (Figure 25A). Through comparative docking with two distinct sets, one optimized for dGMII [52,53] and another tested experimentally in hGMII [104], they revealed a critical species-specificity issue. The MD-relaxed hGMII model at pH 6.6 showed excellent correlation (R^2^ = 0.80) between predicted binding energies and experimental K_i_ values for hGMII-validated inhibitors. Crucially, however, the same model failed to predict dGMII-optimized compound activities (R^2^ = 0.32–0.39), revealing that inhibitors designed using non-human orthologs may not translate to the human target (Figure 25B). MD simulations uncovered the structural basis: hGMII possesses a more spacious active site with key residue differences creating species-specific binding patterns. The study powerfully concludes that computational efforts should prioritize the use of MD-validated human-specific models over non-human orthologs for the rational design of potent and, critically, selective inhibitors.

**Figure 26:**
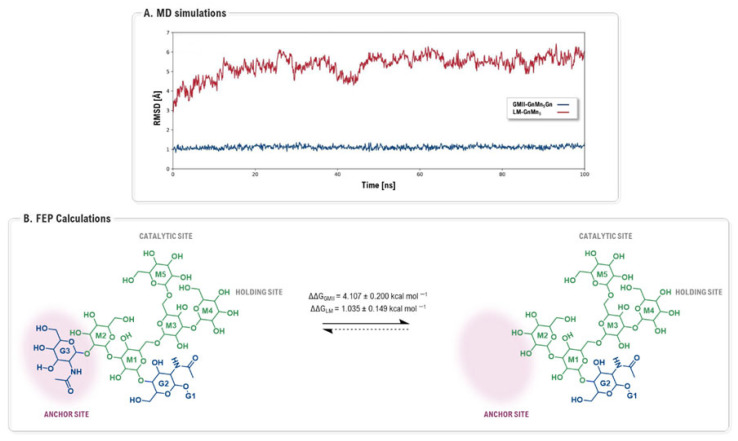
(**A**) MD analysis of ligand stability. The RMSD plot monitors the conformational fluctuations of the terminal N-acetylglucosamine residue (G3) over time. The residue remains tightly bound in the GMII anchor site (blue line) but fluctuates freely in the Lysosomal Mannosidase (LM) (red line). (**B**) FEP calculations quantifying the anchor site contribution. The G3 moiety provides significantly higher stabilization in GMI than in LM revealing a selectivity window of ~3.1 kcal mol^−1^. Reproduced with permission from “Uncovering the Potential of the Anchor Site for Enhancing Golgi Alpha-Mannosidase II Selectivity Using Molecular Dynamics Simulations and Free Energy Calculations”, by Wan and co-workers, published in *Journal of Biomolecular Structure and Dynamics*, 43(13), 7190–7198, 2025. Copyright © Taylor & Francis. Reprinted by permission of Informa UK Limited, trading as Taylor & Francis Group [105].

In 2025, the focus of MD simulations evolved towards not only refining protein models, but also directly quantifying the energetic contributions of specific subsites to inhibitor selectivity. A groundbreaking study by Wan and co-workers used a combination of extensive dynamic simulations and rigorous free energy perturbation (FEP) calculations to investigate the potential of the non-conserved anchor site as a target for achieving selectivity [105]. The MD simulations of the enzyme-substrate complexes provided a clear dynamic picture: the terminal *N*-acetylglucosamine (G3) residue of the substrate remained stably bound within the GMII anchor site, whereas it freely fluctuated when complexed with LMan, which lacks a defined anchor pocket (Figure 26A). This stable anchoring was shown to reduce the overall flexibility of the substrate bound to GMII. Significantly, the subsequent FEP calculations provided a quantitative measure of this phenomenon. The study calculated that the interaction of the G3 residue with the anchor site contributes a significant 4.1 kcal mol^−1^ to the total binding free energy in GMII, compared to a mere 1.0 kcal mol^−1^ in LMan. This calculated energetic difference of ~3.1 kcal mol^−1^ represents a quantitative prediction of the potential gain in selectivity that could be achieved by designing an inhibitor with a moiety that effectively targets this unique anchor site (Figure 26B). This work stands as a prime example of how advanced computational techniques can move beyond qualitative descriptions to provide quantitative, actionable data for the rational design of highly selective inhibitors.

Collectively, these advances underscore the evolution of MD from a retrospective validation tool into a proactive, quantitative engine capable of refining species-specific models and accurately predicting the energetic dividends of targeting unique subsites, thus laying the foundation for the next generation of highly selective GMII inhibitors.

## 4. Overview of Computational Methods

The research discussed in this review relies on a sophisticated hierarchy of computational techniques, ranging from classical structure-based methods to high-level quantum mechanical calculations. To facilitate the understanding of the technical aspects behind these studies, we have categorized the methodologies according to their specific application in the GMII selectivity challenge: structural screening, dynamic sampling, and electronic/energetic quantification. Table 3 provides a concise glossary of these methods, outlining their theoretical basis and their distinct utility in the rational design of GMII inhibitors.

## 5. Overview of Inhibitors and Their Properties

To facilitate the comparison of the potency of the diverse scaffolds explored in the literature, Table 4 provides a comprehensive summary of the inhibitory activities of the compounds discussed in the review. The values for the half-maximal inhibitory concentration (IC_50_) and/or the inhibition constant (*K*i) are included for the differentiation between the Golgi (GMII) and lysosomal mannosidase (LMan) isoforms.

## 6. Conclusions

The comprehensive analysis of the computational strategies reviewed herein demonstrates that high selectivity for Golgi α-mannosidase II (GMII) over its lysosomal counterpart (LManII) and other related glycosidases is no longer a matter of serendipity, but the result of a rational, multidimensional inhibitor design process. Structurally, the most successful trajectory has shifted from modifying the glycone core to exploiting the subtle architectural divergences in peripheral subsites. The functionalization of the central nitrogen atom of the iminosugar scaffold with aryl-alkyl or basic linkers has proven to be one of the most robust strategies, enabling the targeting of non-conserved anchor site through specific interactions, such as π–π stacking with Tyr267 or salt bridges with the Asp270-Asp340 dyad, that are structurally inaccessible in the more open, hydrophilic active site of LManII. This structure-based optimization has culminated in the development of rigid bicyclic scaffolds which, by locking the bioactive conformation and effectively filling these hydrophobic pockets, have yielded the most potent candidates to date, achieving nanomolar affinity and exceptional selectivity indices (SI > 100) suitable for in vivo application. Crucially, this structural complementarity is inextricably linked to “electrostatic selectivity”: by fine-tuning the basicity of the inhibitor, rational design can favor a neutral binding state at the Golgi pH (~6.0), thereby avoiding severe electrostatic repulsions and desolvation penalties that the protonated species suffer in the acidic environment of the lysosome (~4.5). Furthermore, the most recent advances have unveiled that introducing steric strain via substitution at the C-5 position or exploiting fluorine bioisosteres at C-6 of the indolizidine ring can force specific ring distortions that only the GMII active site can accommodate, imposing a prohibitive energetic cost on LManII binding.

Consequently, realizing these complex design principles has required a profound maturation of the in silico spectrum, evolving from simple static models into sophisticated, multi-scale pipelines. While Molecular Docking and Virtual Screening remain foundational for expanding chemical space beyond iminosugars, this review highlights that static complementarity alone is insufficient to capture the nuanced thermodynamics governing GH38 inhibition. The field has therefore established Quantum–mechanical insights, specifically via FMO-PIEDA, SAPT and in situ pKa calculations, as indispensable tools for accurately modeling the polarization effects around the catalytic Zn^2+^ ion and predicting the delicate pH-dependent equilibrium mentioned above. This electronic precision is now synergistically coupled with Molecular Dynamics and Free Energy Perturbation, which have transformed our understanding of the plasticity of the target by validating human-specific homology models and providing rigorous quantitative predictions of the binding free energy associated with targeting specific peripheral hotspots.

However, the realization of the full potential of these predictive workflows is predicated upon the continuous integration of experimental validation. The experimental confirmation of the predicted binding modes, the calculated pKa shifts, and the free energy profiles through biophysical and kinetic assays remains an essential step to ensure the accuracy of the computational models and to guide the iterative refinement of the next generation of highly selective GMII inhibitors. In conclusion, the future of GMII inhibitor discovery relies on the rigorous application of these integrated workflows, bridging the gap between the potency of enzymatic inhibition and the clinical requirement for exquisite selectivity.

## Figures and Tables

**Table 1:** Equivalence of Key Active Site Residues between hGMII and dGMII.

Protein	Residue							
hGMII	His175	Asp177	Trp180	Asp289	Phe291	Tyr354	Asp426	His569
dGMII	His90	Asp92	Trp95	Asp204	Phe206	Tyr269	Asp341	His471

**Table 2:** Total and partial interaction energies (**Δ*E*_I-E_**, **Δ*E*_ring-E_** and **Δ*E*_benzyl-E_** in kcal mol^−1^) and percentage contribution of the benzyl group for different charge forms of inhibitor **19** in complexes with dGMII and JBMan calculated at the MP2//M06-2X level. Data adapted from Kalník and coworkers [50] with additional percentage contribution analysis.

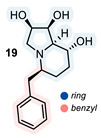	**dGMII**	**Conformation**	**Δ*E*_I-E_**	**Δ*E*_ring-E_**	**Δ*E*_benzyl-E_**	**Benzyl** **Contribution (%)**
	**19^0^-Asp341^−^**	*E*_4_	−615.7	−577.8	−37.9	6.2
		*E*_4_/^3^*E*	−632.0	−593.8	−38.2	6.0
	**19^0^-Asp341^0^**	*E*_4_	−604.9	−568.4	−36.5	6.0
		*E*_4_/^3^*E*	−599.2	−566.9	−32.3	5.4
	**19^+^-Asp341^−^**	*E*_4_	−803.4	−773.2	−30.2	3.8
		*E*_4_/^3^*E*	−793.1	−763.7	−29.4	3.7
	**19^+^-Asp341^0^**	*E*_4_	−746.7	−721.6	−25.1	3.4
		*E*_4_/^3^*E*	−740.1	−715.2	−24.9	3.4
	**JBMan**	**Conformation**	**Δ*E*_I-E_**	**Δ*E*_ring-E_**	**Δ*E*_benzyl-E_**	**Benzyl** **Contribution (%)**
	**19^0^-Asp260^−^**	*E*_4_	−583.2	−562.6	−20.6	3.5
		^3^ *E*	−604.1	−583.7	−20.5	3.5
	**19^+^-Asp268^−^**	*E*_4_	−787.1	−760.5	−26.6	3.4
		^3^ *E*	−789.3	−769.5	−19.8	2.5

**Table 3:** Computational Methodologies Employed in GMII Inhibitor Research.

Methodology	Acronym/*Tools*	Description and Applications in Drug Design	Refs.
Protein Structure Prediction	*AlphaFold*, *Homology*, *Modeling*	Constructs 3D protein models from sequence data using AI or homologous templates	[27,106]
QM/MM Metadynamics	QM/MM	Simulates enzymatic reactions and transition states by combining QM accuracy with MM efficiency	[107]
Ab Initio Metadynamics	QM/MD	Unbiased sampling of small molecule free-energy landscapes to identify stable conformers	[108]
QM-Based QSAR	QSAR/MLR	Correlates biological activity with electronic structure descriptors derived from QM calculations	[109]
Density Functional Theory	DFT	High-accuracy QM method for calculating electronic structure, bond energies, and geometries	[24]
Symmetry-Adapted Perturbation Theory	SAPT	Decomposes intermolecular interaction energy into electrostatic, exchange, induction, and dispersion components	[57]
Fragment Molecular Orbital	FMO/FMO-PIEDA	Fragments large biomolecules to efficiently compute pairwise residue-ligand interaction energies	[25,59]
In situ pKa Prediction	PROPKA	Predicts ionization constants of residues and ligands within the specific protein environment	[61,110]
Molecular Docking	*GOLD*, *FlexX*, *Glide*, *AutoDock*, *DOCK*	Predicts ligand binding poses and estimates affinity using structure-based scoring functions	[111]
Binding Site Detection	*Site Finder*, *DoGSiteScorer*	Identifies and scores potential ligand-binding cavities and allosteric pockets on protein surfaces	[89,90]
Molecular Fingerprinting	*PLIF*, *MACCS*	Encodes molecular structures or interaction patterns into vectors for rapid similarity searching	[112]
Pharmacophore Modeling	-	Defines the spatial arrangement of chemical features essential for ligand-target binding	[113]
Virtual Screening	VS	Automated filtering of large chemical libraries to identify potential bioactive “hits”	[114]
ADMET Filtering	In silico *ADMET*	Predicts pharmacokinetic properties (Absorption, Distribution, Metabolism, Excretion) and toxicity	[115]
Molecular Dynamics	MD (on/off constant pH)	Simulates atomic movements over time to reveal system flexibility and complex stability	[116]
NMR Simulation	*CORCEMA-ST*	Predicts NMR signals (e.g., STD) from structural models to validate binding poses against experimental data	[96]
Electronic Structure Analysis	NCI/NBO	Visualizes and quantifies non-covalent interactions and electron delocalization	[99,100]
Enhanced Sampling MD	REST-RECT	Overcomes energy barriers to efficiently sample rare events and broad conformational spaces	[44]
Free Energy Perturbation	FEP	Rigorously calculates free energy differences between states for high-accuracy affinity prediction	[117]

**Table 4:** Summary of the inhibitory activities (GMII and LMan) of the inhibitors discussed in the review.

Compound	GMII	LMan	Refs.
Swainsonine, **1**	IC_50_ = 4 nM (hGMII) Ki = 5 nM (hGMII)	IC_50_ = 20 nM (hGMII) Ki = 23 nM (hGMII)	[12]
Noeuromycin, **2**	IC_50_ = 20 µM (dGMII)	-	[39]
Mannoimidazole, **3**	Ki = 2 µM (dGMII)	Ki = 20 µM (dLMan)	[40]
Kifunensine, **4**	Ki = 5.2 mM (dGMII)	-	[43]
**5**	Ki = 2 mM (dGMII)	-	[56]
Mannostatin A, **6**	Ki = 0.21 µM(hGMII)	Ki = 0.09 µM (dGMII)	[67]
**8**	64% inhibition (at 1 mM) (hGMII)	80% inhibition (at 1 mM) (hLMan)	[17]
**9**	IC_50_ = 270 µM (GMIIb) Ki = 220 µM (GMIIb)	IC_50_ = 7.5 mM (LManII)	[51]
**10**	IC_50_ = 52 µM (GMIIb) Ki = 50 µM (GMIIb)	IC_50_ = 6.1 mM (LManII)	[51]
**11**	IC_50_ = 55 µM (GMIIb) Ki = 58 µM (GMIIb)	IC_50_ = 7.5 mM (LManII)	[51]
**12**	IC_50_ = 42 µM (GMIIb) Ki = 19 µM (GMIIb)	-	[53]
**13**	IC_50_ = 8 µM (GMIIb) Ki = 4 µM (GMIIb)	18% inhibition (at 1 mM) (LManII)	[52]
**14**	IC_50_ = 9 µM (GMIIb) Ki = 5.5 µM (GMIIb)	27% inhibition (at 1 mM) (LManII)	[52]
**15**	IC_50_ = 450 nM (GMIIb) IC_50_ = 210 nM (AMAN-2) Ki = 160 nM (GMIIb) Ki = 150 nM (AMAN-2)	IC_50_ = 12 µM (LManII) IC_50_ = 18 µM (JBMan) Ki = 3.9 µM (LManII) Ki = 6.5 µM (JBMan)	[49]
**16**	IC_50_ = 120 nM (GMIIb) IC_50_ = 240 nM (AMAN-2) Ki = 65 nM (GMIIb) Ki = 190 nM (AMAN-2)	IC_50_ = 820 nM (LManII) IC_50_ = 320 nM (JBMan) Ki = 380 µM (LManII) Ki = 120 µM (JBMan)	[54]
**17**	IC_50_ = 13.5 µM (GMIIb) IC_50_ = 22 µM (AMAN-2) Ki = 5.2 µM (GMIIb) Ki = 18 µM (AMAN-2)	IC_50_ = 118 µM (LManII) IC_50_ = 78 µM (JBMan) Ki = 98 µM (LManII) Ki = 44 µM (JBMan)	[54]
**18**	IC_50_ = 7.6 µM (GMIIb) IC_50_ = 2.4 µM (AMAN-2)	IC_50_ = 845 µM (LManII) IC_50_ = 1950 µM (JBMan)	[49]
**19**	Ki = 23 nM (AMAN-2)	Ki = 20 µM (JBMan)	[50]
**20**	Ki = 50 µM (hGMII)	Ki = 6.6 µM (hLMan)	[67]
**21**	IC_50_ = 2 mM (dGMII)	-	[68]
**22**	IC_50_ = 14 µM (dGMII)	-	[68]
**23**	IC_50_ = 2 mM (dGMIIb)	-	[56]
**24**	IC_50_ = 5 mM (dGMIIb)	IC_50_ = 5 mM (dLManII)	[55]
**25**	IC_50_ = 200 µM (GMIIb)	IC_50_ = 1930 µM (LManII)	[69]
**26**	IC_50_ = 3 µM (GMIIb)	IC_50_ = 70 µM (LManII)	[70]
**27**	IC_50_ = 0.3 µM (hGMII) Ki = 24 nM (hGMII)	-	[72]
**28**	IC_50_ = 0.5 µM (hGMII) Ki = 31 nM (hGMII)	-	[72]
**29**	Ki = 97 nM (hGMII)	-	[12]
**30**	Ki = 350 nM (hGMII)	Ki = 4.72 µM (hLMan)	[12]
**31**	IC_50_ = 52 nM (hGMII) Ki = 43 nM (hGMII)	IC_50_ = 7.2 µM (hLMan)	[12]
**32**	IC_50_ = 0.50 µM (JBMan)	-	[20]
**33**	IC_50_ = 0.78 µM (JBMan)		[20]
**34**	IC_50_ = 0.44 µM (JBMan)	-	[20]
**35**	89% inhibition (at 1 mM) (JBMan)	-	[83]
**36**	IC_50_ = 0.5 µM	-	[85]
**37**	IC_50_ = 217 µM (dGMII)	-	[88]
**38**	*K*i = 0.249 µM	-	[91]
**39**	*K*i = 265 µM	-	[91]
**40**	IC_50_ = 175 µM (GMIIb)	IC_50_ = 2450 µM (LManII)	[94]
**41**	IC_50_ = 1.4 µM (GMIIb)	IC_50_ = 230 µM (LManII)	[94]
**42**	IC_50_ = 95 nM (GMIIb)	IC_50_ = 380 µM (LManII)	[94]
**43**	IC_50_ = 25 nM (GMIIb)	-	[94]
**44a**	IC_50_ = 5.3 µM (JBMan) IC_50_ = 3.7 µM (GMIIb)	IC_50_ = 173 µM (LManII)	[98]
**45a**	IC_50_ = 14.8 µM (JBMan) IC_50_ = 5.3 µM (GMIIb)	IC_50_ = 865 µM (LManII)	[98]
**46a**	IC_50_ = 1.2 µM (JBMan) IC_50_ = 0.7 µM (GMIIb)	IC_50_ = 780 µM (LManII)	[98]
**46b**	IC_50_ = 10.5 µM (JBMan) IC_50_ = 28.5 µM (GMIIb)	IC_50_ = 975 µM (LManII)	[98]

## Data Availability

No new data were created or analyzed in this study.

## Abbreviations

Acc	acceptor
ADMDP	aminodeoxy-2,5-dideoxy-2,5-imino-D-mannitol/aminodeoxy-DMDP
ADMET	Absorption, Distribution, Metabolism, Excretion and Toxicity
AMAN-2	*Caenorhabditis elegans* Golgi *α*-mannosidase II
Aro	aromatic
bLMan	bovine lysosomal *α*-mannosidase
CADD	Computer-Aided Drug Design
CAZyme	Carbohydrate-Active enZyme
CGS	Computation-Guided Synthesis
DAB	1,4-dideoxy-1,4-imino-D-arabinitol
DFT	Density Functional Theory
dGMII	*Drosophila melanogaster* Golgi *α*-mannosidase II
DIM	1,4-dideoxy-1,4-imino-D-mannitol
DMDP	2,5-dideoxy-2,5-imino-D-mannitol
Don	donor
FEL	Free-Energy Landscape
FEP	Free Energy Perturbation
FMO	Fragment Molecular Orbital
G3	*N*-acetylglucosamine-3/GlcNAc-3
GMI	Golgi *α*-mannosidase II
GTs	glycosyltransferases
hGMII	Human Golgi *α*-mannosidase II
Hyd	hydrophobic
JBMan	*Jack Bean α*-mannosidase
LMan	lysosomal *α*-mannosidase
M3	mannose-3
M4	mannose-4
M5	mannose-5
MACCS	molecular access system
MD	Molecular Dynamics
MIm	mannoimidazole
MM	Molecular Mechanics
MSA	mannostatin
NBO	Natural Bond Orbital
NCI	Non-Covalent Interactions
NPICC	Natural Product-Inspired Combinatorial Chemistry
NPs	natural products
QM	Quantum Mechanics
QSAR	Quantitative Structure-Activity Relationship
OCI	oxocarbenium ion character
PIEDA	Pair Interaction Energy Decomposition
PLIF	Protein-Ligand Interaction Fingerprints
REST-RECT	Replica Exchange with Solute Tempering and Collective-Variable Tempering
SAPT	Symmetry Adapted Perturbation Theory
SAR	Structure-Activity Relationship
STD	Saturation Transfer Difference
TS	Transition State
VS	Virtual Screening
